# Dimeric DNA Aptamers for the Spike Protein of SARS‐CoV‐2 Derived from a Structured Library with Dual Random Domains

**DOI:** 10.1002/smtd.202401600

**Published:** 2024-12-20

**Authors:** Ryan Amini, Jian Ma, Zijie Zhang, Qing Wang, Jimmy Gu, Leyla Soleymani, Yingfu Li

**Affiliations:** ^1^ Department of Biochemistry and Biomedical Sciences McMaster University 1280 Main Street West Hamilton ON L8S 4K1 Canada; ^2^ Department of Engineering Physics McMaster University 1280 Main Street West Hamilton ON L8S 4K1 Canada; ^3^ Michael G. DeGroote Institute of Infectious Disease Research McMaster University 1280 Main Street West Hamilton ON L8S 4K1 Canada; ^4^ School of Biomedical Engineering McMaster University 1280 Main Street West Hamilton ON L8S 4K1 Canada

**Keywords:** aptamer, COVID‐19, diagnostics, molecular recognition, structured library

## Abstract

Multimeric aptamer strategies are often adopted to improve the binding affinity of an aptamer toward its target molecules. In most cases, multimeric aptamers are constructed by connecting pre‐identified monomeric aptamers derived from in vitro selection. Although multimerization provides an added benefit of enhanced binding avidity, the characterization of different aptamer pairings adds more steps to an already lengthy procedure. Therefore, an aptamer engineering strategy that directly selects for multimeric aptamers is highly desirable. Here, an in vitro selection strategy is reported on using a pre‐structured DNA library that forms dimeric aptamers. Rather than using a library containing a single random region, which is nearly ubiquitous in existing aptamer selections, the library contains two random regions separated by a flexible poly‐thymidine linker. Following sixteen rounds of selection against the SARS‐CoV‐2 spike protein, a relevant model target protein due to the COVID‐19 pandemic, the top aptamers displayed superb affinity with *K*_D_ values as low as 150 pM. Further analysis reveals that each random region functions as a distinct binding moiety and works together to achieve higher affinity. The demonstrated strategy provides an accelerated method to obtain high‐affinity aptamers, which may prove useful in future aptamer diagnostic and therapeutic applications.

## Graphical abstract

Dimeric in a single go: A novel DNA aptamer library has been designed to yield dimeric aptamers in a single in vitro selection. Tested against the SARS‐CoV‐2 spike protein, this dual random domain (DRD) library with two 25‐nucleotide random regions and a 20‐nucleotide poly‐thymidine linker produced high‐affinity dimeric aptamers with *K*_D_ values as low as 0.15 nM. This streamlined approach accelerates the discovery of dimeric aptamers for diagnostic and therapeutic applications.

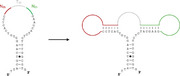

## 1 Introduction

Nucleic acid aptamers are single‐stranded oligonucleotides that can fold into 3D structures for target binding.^[^ ^1^, ^2^ ^]^ Using an in vitro process known as Systematic Evolution of Ligands by Exponential Enrichment (SELEX), many aptamers have been isolated for ions, small molecules, proteins and cells.^[^ ^3^, ^4^ ^]^ Their ability to recognize targets precisely allows them to serve as molecular recognition elements for diagnostic and therapeutic applications.^[^ ^5^ ^]^

An important characteristic of aptamers is the strength of the binding interaction between the nucleic acids and the target, otherwise known as the binding affinity. This is typically measured using the dissociation constant (*K*_D_), which quantifies the equilibrium between the bound and unbound states of the aptamer‐target complex.^[^ ^6^ ^]^ The *K*_D_ value represents the concentration of the target at which half of the aptamer molecules are bound to the target, providing a precise metric for assessing the binding strength. Lower *K*_D_ values indicate higher affinity, signifying a more stable and tighter interaction between the aptamer and its target molecule.

Most aptamers in literature possess a respectable nanomolar binding affinity, which is akin to similar biomolecules such as antibodies.^[^ ^7^, ^8^ ^]^ However, it can be difficult to push beyond this level of affinity for a number of reasons. First, most SELEX experiments produce monomeric‐binding aptamers that only bind to one epitope or subunit on the target. Second, most selections are performed, on average, with 40 nucleotides in the random region. This is typically done to preserve the sequence space (i.e., the total number of possible sequences generated for a given number of nucleotides). However, the use of these shorter random regions prevents the generation of more complex secondary structures, further limiting the interaction interface between the aptamer and the target.^[^ ^9^ ^]^

To mitigate this issue and increase binding affinity, many aptamer research groups use a multivalent strategy post‐selection.^[^ ^10^, ^11^, ^12^, ^13^ ^]^ Multivalent interactions occur when a single biomolecule simultaneously forms several binding interactions with its respective target.^[^ ^14^ ^]^ This phenomenon is commonly observed in biology when precise molecular recognition is needed (e.g., antibodies bind tightly to multiple sites on their respective antigens using their Y‐shape scaffold).^[^ ^15^, ^16^ ^]^ With more than one ligand‐binding site, the binding site's local concentration increases, as does the probability of an interaction.^[^ ^17^ ^]^ In addition to increasing the rate of association, multivalency also reduces the rate of dissociation. If one binding site temporarily dissociates, the other sites remain bound, maintaining the overall complex and facilitating quick rebinding of the dissociated site. This results in a more stable and tighter interaction compared to monovalent binding. Therefore, not surprisingly, the strategy of multivalency and multimerization has been applied to aptamer ligands.^[^ ^18^, ^19^, ^20^, ^21^ ^]^ For example, our group has had recent success in engineering both dimeric and trimeric aptamers for the SARS‐CoV‐2 spike (S) protein (a homotrimeric protein) that show remarkable improvement in affinity when compared to the monomeric aptamer substituent.^[^ ^22^, ^23^, ^24^ ^]^

Yet, this approach to constructing multimeric and multivalent aptamers can be a tedious trial‐and‐error process. It often necessitates finding two aptamers from a diverse pool that (1) bind to different sites or subunits on the same target and (2) work well in tandem.^[^ ^21^ ^]^ Additionally, the process of characterizing multimeric aptamers adds more time to an already lengthy selection procedure. It comes as no surprise that several aptamer research groups have tried to solve this problem by directly selecting for multimeric aptamers. Adopting such a strategy would save weeks in the process and eliminate the characterization steps of aptamer multimerization (i.e., analyzing sequences, characterizing top aptamers, and finding suitable aptamer pairs in a trial‐and‐error process). As an example, Zhou et al., in 2019, fixed two separate random regions between a self‐folding two‐helix tile, achieving aptamers with a femtomolar affinity for thrombin.^[^ ^25^ ^]^ More recently, Tang et al. developed a DNA framework library that mimicked the Y‐shaped scaffold of an antibody.^[^ ^26^ ^]^ Their resulting antibody‐mimicking multivalent aptamers (Amap) showed great binding affinity and cooperativity to the protein target. Therefore, the idea of directly selecting for multivalent and multimeric aptamers is a valuable approach that could significantly streamline the development of high‐affinity aptamers.

Whilst the two listed studies of bivalent aptamer selections demonstrate innovative approaches in achieving high‐affinity aptamers, each method has its own set of limitations. In the former example from Zhou et al., an exceptionally low *K*_D_ value was achieved. However, the paranemic crossover method requires a complicated design, which may limit the streamlining for other targets. Additionally, the short distance between the two random loops in the library poses a challenge for selecting bivalent aptamers against larger target molecules. In the latter example from Tang et al., the *K*_D_ of the Amap aptamers did not exceed the nM range despite their intuitive antibody‐mimicking approach. Moreover, both of these selection strategies also used a rigid linker, which can be less adaptable to variations in the target shape. Flexible linkers, such as the poly‐thymidine (poly‐T) linker, can be more advantageous for certain targets because they allow for greater freedom of movement and help the aptamer adapt to the 3D shape of the target.^[^ ^20^ ^]^ To the best of our knowledge, no group in the functional nucleic acid literature has directly selected for multimeric aptamers using a flexible poly‐nucleotide linker.

Herein, we report on the selection of DNA aptamers using a novel library design that directly selects for dimeric aptamers with a flexible linker. Termed the dual random domain (DRD) library, our library contains two 25‐nucleotide random regions that are separated by a pre‐structured 20‐nucleotide poly‐T linker. Together, with sufficient separation from the poly‐T linker, both random regions can recognize a separate subunit of a protein target and emulate the dual‐arm binding effect seen in dimeric aptamers. Sixteen rounds of selection were conducted against the SARS‐CoV‐2 S protein, a model protein target. The resulting DRD aptamers exhibit a very high binding affinity for the S protein (*K*_D_ = 150 pM), and follow‐up characterization experiments clearly demonstrate the “dimeric‐like” quality of the aptamers. Therefore, the DRD library design represents an attractive strategy for future selection experiments that require a rapid generation of high‐affinity dimeric aptamers for dimeric or multimeric protein targets.

## 2 Results and Discussion

### 2.1 Library Design and Aptamer Selection

**Figure 1:**
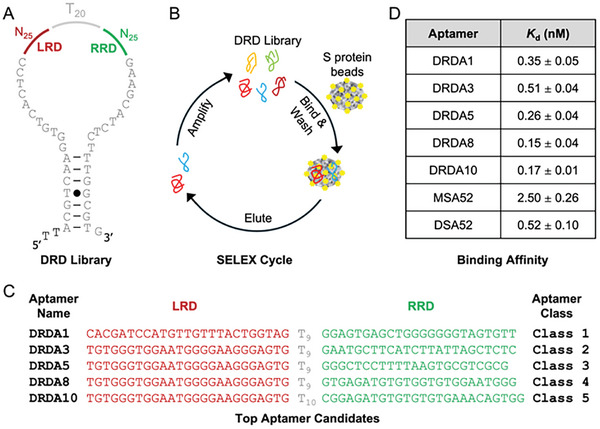
Selection of dimeric aptamers from a dual random domain (DRD) library. A) Library design. The library contains two 25‐nt random domains labeled LRD (left random domain) and RRD (right random domain) separated by a T20 (20‐thymidine) linker. The flanking primer sequences are purposely arranged into a stable hairpin stem previously shown to be effective in generating diverse aptamers. B) SELEX strategy. Each SELEX cycle has three key steps: 1) binding of the DNA pool with S protein‐coated magnetic beads and washing to remove non‐aptameric sequences, 2) eluting aptamer candidates from the beads and 3) amplification with PCR to generate the next DNA pool. C) Top 5 aptamer candidates named DRDA1, 3, 5, 8 and 10 representing the populous members of DRD aptamer class 1–5, respectively. D) Binding affinity (measured by the *K*_D_ value) of the top 5 aptamers. *K*_D_ values listed as mean ± SD (*n* = 3).

The DNA library is named the dual random domain, or DRD for short (all synthetic oligonucleotides utilized in this study are provided in Table S1, Supporting Information). As illustrated in **Figure** **1**A, the DRD library was designed with two 25‐nucleotide fully randomized regions, one region on the left indicated in red (LRD) and one on the right indicated in green (RRD). These two regions are connected by a 20‐nucleotide poly‐T linker (T20), indicated in grey. The design also used a previously established pairing element where the flanking primers were arranged to create a stable hairpin stem.^[^ ^22^ ^]^ This was done for two reasons: (1) to ensure that the primers do not play an important role in target binding, and (2) to emulate many other high‐affinity DNA aptamers, which also possess hairpin stems.^[^ ^27^, ^28^, ^29^ ^]^ Regarding the poly‐T linker, a 20‐nucleotide length was used because our previous S protein dimeric aptamer research showed that a 20 nucleotide length enables higher binding affinity of dimeric aptamers.^[^ ^23^ ^]^ Finally, each random region was chosen to consist of 25 nucleotides to preserve a sufficient nucleotide length to enable large secondary structures, such as loops and hairpins. The choice of 25 nucleotides was also guided by our previous work with truncated SARS‐CoV‐2 aptamers, which demonstrated that secondary structure elements of this length were sufficient for high affinity binding. Extending the regions beyond 25 nucleotides was avoided to prevent issues associated with longer libraries, including limitations in adequately covering sequence space and an increased likelihood of PCR artifacts during selection rounds, which can compromise the integrity of the library. Magnetic bead‐based selection was used to isolate aptamers from the library. Histidine‐tagged trimeric S protein of the Omicron BA.5 subvariant of SARS‐CoV‐2 was conjugated onto nickel‐nitrilotriacetic acid (Ni‐NTA) modified magnetic beads. The BA.5 S protein was chosen as the selection target because it was the latest available protein of the Omicron subvariant at the time.

Aptamer selection was carried out using the strategy shown in Figure 1B, which illustrates a typical SELEX cycle with magnetic beads coated with the BA.5 S protein. The DNA library was incubated with the beads; unbound sequences were removed through washing, and the bound sequences were eluted and then amplified via PCR to generate an enriched pool for the following round. Two sets of PCR (polymerase chain reaction) were conducted per round. The first set was completed with the forward primer and reverse primer. The second set was instead completed with the forward primer and reverse blocked primer, which contains the sequence of the reverse primer and a non‐amplifiable hexa‐ethylene glycol linker. This design allowed us to get single‐stranded sense DNA (ssDNA) by running the PCR products on a denaturing (8 M urea) polyacrylamide gel electrophoresis (dPAGE).^[^ ^22^ ^]^

The selection pressure was gradually increased to create more stringent conditions by decreasing the amount of protein and library (Table S2, Supporting Information) to select aptamers with the highest affinity from the library. More specifically, the selection began with a 10,000 nm DNA library and 4,000 nm of BA.5 S protein. Most of the following rounds, with the exception of rounds 4–6 and 10–12, had a near twofold reduction in concentration to facilitate the selection of aptamers with excellent binding affinity. A total of 16 rounds of selection were completed; at round 16, the library and the target concentrations were reduced to 2.5 and 1.25 nm, respectively, representing 400‐ and 320‐fold reductions over the initial library and target concentrations.

Throughout the selection, the binding affinity of the library pools of rounds 0, 6, 8, 10, 12, 14, and 16 were evaluated by electrophoretic mobility shift assay (EMSA). The DNA libraries were labelled with ^32^P at the 5′ end, and then incubated with the S protein to allow binding to occur. Aptamer‐protein solutions were then loaded onto a 10% native PAGE to allow for the separation of the aptamer‐target complexes and the free labelled DNA sequences. In principle, the aptamer‐target complexes are larger and move slower on the native PAGE, compared to unbound DNA.^[^ ^30^ ^]^ The aptamer bound fraction, as a function of the target protein concentration, was plotted to derive corresponding *K*_D_ values of the pools. The EMSAs shown in Figure S1 (Supporting Information) confirmed that the selection was successful, in that the enriched pools were binding to the S protein. Of importance was the significant jump in binding affinity from round 6 (14 nm) to round 8 (1.4 nm), likely attributed to the increased selection stringency. An increase was also observed for later pools, but the level of binding plateaued in later rounds, with *K*_D_ values ranging between 100–600 pm for the enriched pools of round 10–16.

High‐throughput sequencing was then completed with all 16 pools using a previously described protocol.^[^ ^31^ ^]^ The top 50 unique sequences are listed in Table S3 (Supporting Information); Each sequence is named DRDAX, where DRDA stands for Dual Random Domain Aptamer, and X is the numeral that represents the ranking of the sequence in the round 16 pool.

The top 50 sequences occupied 30.143% of pool 16, with the percentage in the pool varying from 2.7968% (the top‐ranked sequence) to 0.1975% (the 50^th^ ranked sequence). These 50 sequences were then sorted into 14 different classes (Table S4, Supporting Information). Class 2 has the most members (having 8 members) within the top 50 sequences, followed by Classes 1 and 3 (each with 5 members), by Classes 4, 5, 6 and 7 (4‐member family), by Classes 8, 9, and 10 (3‐member class), by Classes 11, 12 and 13 (2 members each), and Class 14 (1 member only). The members of each family share identical or nearly identical nucleotide sequences except for the original T20 element, which was significantly reduced from the original 20 T‐units to a size varying between 8–12 T‐units (this phenomenon will be further investigated later in the report).

In terms of percentage in pool 16, Class 1 is the most dominant class (6.7985% in pool 16), which is closely followed by Class 2 (5.7558%). The occupancy of the other classes varies between 3.7607% (Class 3) to 0.2243% (Class 14).

To our knowledge, a single SELEX experiment rarely produces this many aptamer classes. This finding suggests that the structured library with dual random domains represents an effective way of discovering very diverse aptamers for a protein target with multiple subunits like the S protein of SARS‐CoV‐2.

Interestingly, some of the top 14 classes share the same left random domain (Table S5, Supporting Information; LRD: left random domain) or the same right random domain (Table S6 (Supporting Information); RRD: right random domain). Most notably, Classes 2, 3, 4, 5 and 7 share the same sequence named LRD2, making this left random domain the most dominant LRD (accounting for 16.2554% in the top 50 sequences; Table S5, Supporting Information), outcompeting LRD1 (6.7985%; Table S5, Supporting Information) as the LRD in the Class 1 sequence. Two other left random domains, namely LRD3 and LRD7, are each shared by two different classes. For the right random domains, RRD5 and RRD10 appear three times and twice respectively in the top 14 classes (Table S6, Supporting Information). RRD1 is the most abundant RRD (accounting for 6.7985% in the top 50 sequences; Table S6, Supporting Information), followed closely by RRD2 (5.7558%) and RRD5 (5.5270%). Intriguingly, Class 5 is the most unique class as it contains both an LRD (LRD2) that appears in 5 different classes and an RRD (RRD5) that is observed in three different classes.

### 2.2 Assessment of Binding Affinity of Top 5 Aptamer Classes

Typically, following an aptamer selection, the top‐ranking unique sequences from the final pool are tested for their affinity. However, as discussed in the last section, the top 50 individual sequences in pool 16 belong to 14 different classes each sharing the same LRD and RRD but differing only in the poly‐T linker length (Table S4, Supporting Information). Based on this observation, we decided to test the top 5 classes using the most populous sequence of each class as its representative. Accordingly, we tested DRDA1, 3, 5, 8, and 10 as the representatives for classes 1, 2, 3, 4, and 5, respectively (the sequences of LRD, RRD and the T‐linker elements of these aptamers are provided in Figure 1C).

The binding affinity of these 5 aptamers was measured using the standard dot blot assays, a common and simple technique used to test the affinity of DNA‐protein interactions.^[^ ^32^, ^33^, ^34^ ^]^ Compared to other binding assessment methods such as SPR, dot blot assays offer a simpler, more cost‐effective method for high‐throughput initial screening, accommodating a variety of sample types and providing clear, qualitative insights into DNA‐protein binding affinities.^[^ ^35^ ^]^ The dot‐blot has been well‐established in our previous studies on SARS‐CoV‐2 S‐protein aptamers,^[^ ^8^, ^22^, ^23^, ^24^, ^36^ ^]^ allowing for consistent and comparable results across our research on aptamer‐protein interactions. Figure S2A (Supporting Information) displays a representative dot blot assay for DRDA1 and DRDA8; Figure S2B (Supporting Information) presents the corresponding binding curves used to derive the *K*_D_ values for these two aptamers. The table in Figure 1D summarizes the *K*_D_ values of all five DRD aptamers, along with the *K*_D_ values of two aptamer controls – the monomeric aptamer MSA52 and its dimeric aptamer derivative named DSA52 – for comparison. Note that DSA52 is designed to contain two MSA52 sequence elements linked with a T_20_ linker (see its sequence in Table S1, Supporting Information).

All five aptamers exhibit excellent binding affinity, with *K*_D_ values below 1 nM (Figure 1D). DRDA8 produced the highest binding affinity with a *K*_D_ value of 0.15 nm (150 pm). They are followed by DRDA10 (0.17 nm), DRDA5 (0.26 nm), DRDA1 (0.35 nm) and DRDA3 (0.51 nm). All DRD aptamers show much better affinity over MSA52 (*K*_D_ = 2.50 nm). The *K*_D_ value of the best DRD aptamer – DRDA8 – is 17‐fold lower than MSA52. In comparison to DSA52, which was determined to have a *K*_D_ value of 0.52 nm, all five DRD aptamers exhibit a better affinity. DRDA8 in particular has a *K*_D_ value that is threefold lower than DSA52. Thus, the DRD library approach can lead the isolation of aptamers with dimeric‐like affinity for the S protein in a single selection.

### 2.3 Predicted Secondary Structures of Top 5 Aptamer Classes

**Figure 2:**
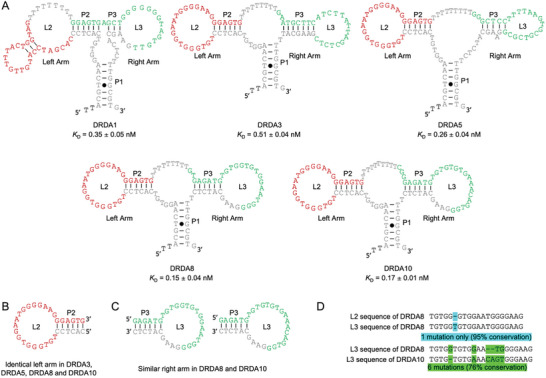
A) Secondary structures predicted for DRDA1, DRDA3, DRDA5, DRDA8, and DRDA10 (representing DRD aptamer classes 1–5, respectively), along with their respective *K*_D_ value (mean ± SD, *n* = 3). B) The identical left arms observed in DRDA3, DRDA5, DRDA8, and DRDA10. C) Highly similar right arms observed in DRDA8 and DRDA10. D) Nucleotide conservation analysis of the left loop L2 sequence of DRDA8 and its right loop L3 sequence (top) and the right loop L3 sequences of DRDA8 and DRDA10 (bottom).

The dominant secondary structures of DRDA1, 3, 5, 8, and 10 that were predicted using mfold are shown in **Figure** **2**A. All 5 aptamers appear to adopt a 3‐way junction‐based structural arrangement where P1 is the pre‐arranged duplex element, P2 and P3 being evolved either from one random domain (in the case of DRDA1) or from two random domains (in the cases of DRDA3, 5, 8 and 10). Each P2 is linked to a loop sequence named L2 and together the P2‐L2 element acts as the left arm of the structure. Similarly, each P3 is linked to its loop L3, and the P3‐L3 element functions as the right arm of the structure. These structural features give all top 5 aptamers very similar secondary structure appearances. Interestingly, four of the five aptamers (i.e., DRDA3, DRDA5, DRDA8, and DRDA10) share the identical P2‐L2 left arm (Figure 2B), suggesting this hairpin‐shaped arm is a highly important sequence and structural element for target binding.

Careful inspection of the left arm loop L2 and the right arm loop L3 in DRDA8 led to the discovery that the L2 and L3 sequences are nearly identical except for the extra T nucleotide in L3 (thus the two sequences share 95% nucleotide conservation; Figure 2D – top); in other words, this highly conserved sequence element appears twice in the sequence of DRDA8. This observation suggests that DRDA8 may function as a homodimer.

Another intriguing discovery is the observation that the sequences of the right arms of DRDA8 and DRDA10 are very similar (Figure 2C), as sequence alignment reveals that the L3 sequences of DRDA8 and DRDA10 share 76% nucleotide conservation (6 mutations are observed in the 21‐nt right loop sequence; see Figure 2D – bottom). This finding suggests that DRDA8 and DRDA10 are closely related. The similar *K*_D_ values further support this premise. Given these observations, we chose to focus on DRDA8 and DRDA10 as the aptamer candidates for further characterization in the remainder of this work.

### 2.4 Binding of a Pseudotyped Omicron BA.5 SARS‐CoV‐2 Lentivirus by DRDA8 and DRDA10

**Figure 3:**
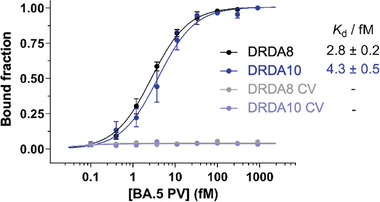
Assessment of binding affinity of DRDA8 and DRDA10 to pseudotyped lentiviruses (PV) engineered to display the Omicron BA.5 S protein of SARS‐CoV‐2. Binding curves used to determine *K*_D_ values (mean ± SD, *n* ≥ 3) for DRD aptamers. CV: lentivirus that lacks the S protein. An estimated 100 pM of labelled DNA aptamer was incubated with BA.5 S protein of concentrations ranging from 900‐0.1 fM for 10 min. Following incubation, the aptamer‐protein mixtures were subjected through dot blot filtration and the membranes were subsequently imaged. Bound fractions were quantified and plotted to obtain *K*_D_ values.

We then tested the binding of DRDA8 and DRDA10 to pseudotyped lentiviruses (PV) expressing the Omicron BA.5 S protein. As mentioned in our previous papers, these lentiviruses resemble that of SARS‐CoV‐2 but cannot replicate themselves beyond cell entry; they are, therefore, a safe and adequate substitute for SARS‐CoV‐2.^[^ ^37^ ^]^ Each viral particle of SARS‐CoV‐2 carries multiple trimeric S proteins, and thus, an enhanced affinity can be expected. The same lentivirus that lacks the Omicron BA.5 S proteins on its surface was used as a control (CV). Dot‐blot assays were once again used to measure the affinity against the PV and CV, and the results are presented in **Figure** **3**. Both DRDA8 and DRDA10 could bind and recognize the PV, but did not demonstrate any binding to the CV. The *K*_D_ values of DRDA8 and DRDA10 for the PV are 2.8 fM and 4.3 fM, respectively. If we compare this approximation to our previous dimeric aptamer for the SARS‐CoV‐2 S protein, which had a binding affinity for PV in the low pM range,^[^ ^23^ ^]^ we can conclude that the DRD aptamers once again possess an affinity that exceeds our previous dimeric aptamers.

### 2.5 Divalent Characterization of DRDA8 and DRDA10

After confirming the “dimeric‐like” affinity of DRD aptamers for the S protein and the pseudovirus, the next logical step was to validate whether these aptamers possessed characteristics similar to typical dimeric aptamers. We began by further analyzing the secondary structure of DRDA8 and DRDA10. Each aptamer contains 3 paired elements (P1, P2, P3) and 4 unpaired elements (SS12, L2, SS23, and L3). Interestingly, the folded elements produce a structure that resembles a dimeric, dual‐arm shape, where each random region is clearly defined by its own monomeric stem and loop structure.

**Figure 4:**
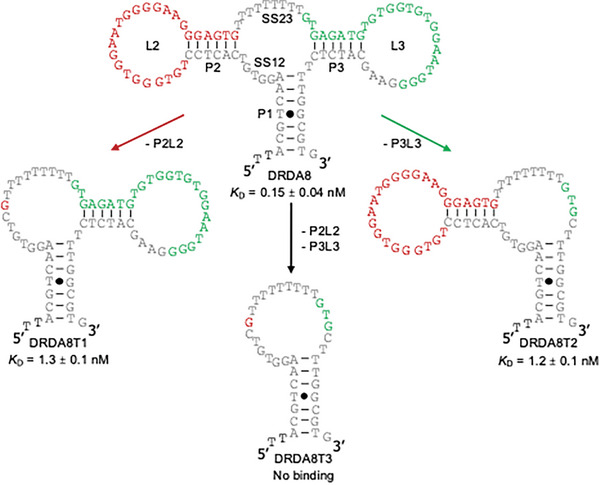
Truncation analysis of DRDA8. Secondary structure and the binding affinity of the full‐length DRDA8 and mutants with truncated domains. *K*_D_ values listed as mean ± SD (*n* ≥ 3).

If the DRD aptamers were to act like dimeric aptamers, we hypothesized that the affinity of each binding arm on its own would show a significant reduction in affinity when compared to the full‐length structure with two arms. To test this, several truncated mutants of DRDA8 were analyzed via dot blot assays for the binding activity to the Omicron BA.5 S protein (**Figure** **4**). Truncation 1 (i.e., mutant aptamer named DRDA8T1) has the structural elements of the first binding arm removed (P2 and L2), Truncation 2 (DRDA8T2) has the structural elements of the second binding arm removed (P3 and L3), and Truncation 3 (DRDA8T3) has both binding arms removed (P2 & L2 and P3 & L3). In DRDA8T1 and DRDA8T2, a near tenfold loss in binding activity is observed (DRDA8T1: *K*_D_ = 1.3 nm, DRDA8T2: 1.2 nm) when compared to the full‐length aptamer (*K*_D_ = 0.15 nm). DRDA8T3 suffers an even greater loss in activity, with no binding observed. These results suggest that both random regions and binding arms are imperative, and they work in cohesion to provide better avidity and affinity to the target, similar to any other dimeric aptamer.

Although not related to the binding arms, the hairpin primers (P1) were also analyzed in Truncation 4 (DRDA8T4) and Truncation 5 (DRDA8T5), with a partial and complete removal, respectively (Figure S3, Supporting Information). In both truncated mutants, the binding activity is still retained (DRDA8T4: *K*_D_ = 0.25 ± 0.09 nm, DRDA8T5: 0.12 ± 0.03 nm), suggesting that the primers play no significant role in target recognition. The similar binding activity observed for the truncated mutants DRDA8T4 and DRDA8T5 compared to the full‐length aptamer DRDA8 suggests that these shorter sequences may offer advantages for practical applications, such as improved stability, reduced synthesis costs, and potentially enhanced binding efficiency due to their compact structure. The same five truncations were performed against DRDA10, and similar trends were observed with the binding arms and hairpin primers (Figure S4, Supporting Information).

To further test the dimeric binding performance of the DRD aptamers, another two mutants are designed to verify the role of the two arms. In the experiment, two DRDA8 mutants were generated with the loop sequences of either binding arm completely scrambled at 100% randomization (L2 Scramble and L3 Scramble). For example, the original L2 sequence of DRDA8 with a sequence of TGTGGGTGGAATGGGGAAG was randomly scrambled to GGTGAATTTGGGGAGGAGG while keeping the same base ratios. The binding of these sequences to the Omicron BA.5 S protein was then analyzed via dot blot analysis (Figure S5, Supporting Information). Similar to the truncation assays, a clear reduction in affinity is observed when one of the two loops is scrambled (*K*_D_ = 1.6 ± 0.4 nm and 2.6 ± 0.8 nm, respectively). Taken together, the truncation and scramble sequence assessments suggest that the two arms in DRDA8 and DRDA10 act synergistically for target recognition, similar to how other high‐performing dimeric aptamers operate.

**Figure 5:**
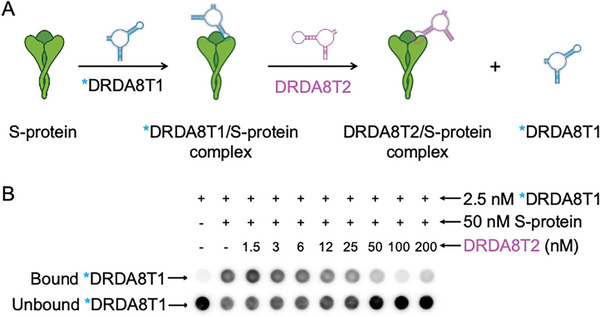
Competition between DRDA8T1 and DRDA8T2 for binding to the S protein. A) Assay schematic. Radioactive DRDA8T1 is allowed to bind fully to BA.5 S protein before competition with DRDA8T2. B) Dot‐blot assay results. A 50 nm solution of BA.5 S protein was incubated with 2.5 nm radioactive (*) DRDA8T1, followed by addition of 1.5–200 nm nonradioactive DRDA8T2.

Another important area of investigation was to assess whether the DRD aptamers were behaving similarly to heterodimers or homodimers.^[^ ^21^ ^]^ Heterodimers, composed of different aptamer ligands, typically recognize distinct epitopes on a multimeric protein. On the other hand, homodimers, which are composed of the same two aptamer ligands, will recognize and bind to the same epitope on differing subunits. To test whether each binding arm recognized a distinct epitope on the Omicron BA.5 S protein, we conducted a competition assay that used non‐radioactive DRDA8T2 to compete with radioactive DRDA8T1. We first incubated radioactive DRDA8T1 under the condition that DRDA8T1 was near‐fully bound to the S protein. Then, non‐radioactive DRDA8T2 was added at varying concentrations to allow for competition. The results in **Figure** **5** indicate that DRDA8T2 competed strongly with DRDA8T1. Initially, DRDA8T1 binds tightly to the S protein at relatively low amounts of competing DRDA8T2. However, as the concentration of DRDA8T2 increased to ≈50 nm, radiolabeled DRDA8T1 was clearly outcompeted by non‐labeled DRDA8T2.

The reverse assay with labelled DRDA8T2 and non‐labelled DRDA8T1 is provided in Figure S6 (Supporting Information). Competition at higher competing aptamer concentrations was observed once again. Overall, the competition in these results strongly suggests that the two arms of DRDA8 act as homodimers, recognizing the same epitopes on differing subunits of the trimeric S protein.

### 2.6 T‐Linker Shortening Analysis

**Figure 6:**
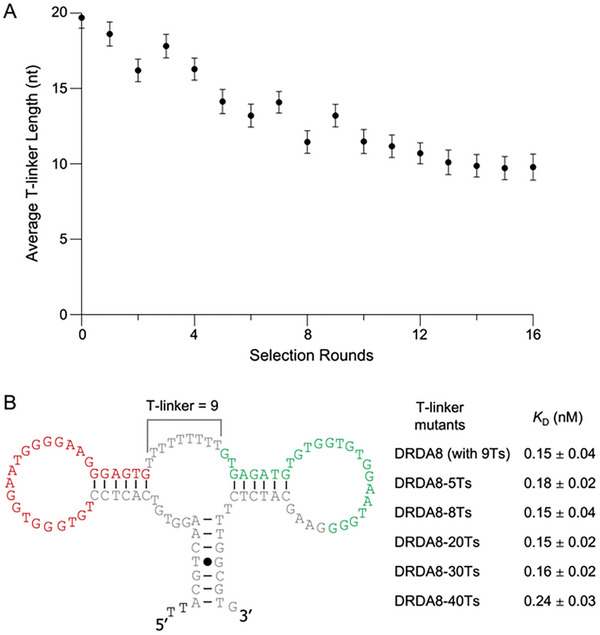
DRD aptamer poly‐T linker analysis. A) Trend of average poly‐T linker length from round 0 to round 16. Linker length decreased from an average of 19.5 nucleotides to 9.8 nucleotides. B) Binding affinity of DRDA8 (which has a T‐linker length of 9) and its mutants with extended or shortened T‐linker lengths. *K*_D_ values listed as mean ± SD (*n* = 3).

One of the more interesting phenomena from the selection was that the poly‐T linker, which was initially designed as 20 nucleotides in the library, gradually decreased to 10 nucleotides in the final selection round (**Figure** **6**A). Our initial assumption was that this was due to a PCR bias, where shorter sequences with deletion mutations were favored by PCR and were amplified at a higher rate than the longer sequences.^[^ ^38^ ^]^ However, we also considered that this reduction in length could also be evolutionarily driven, where the aptamers preferred a specific T‐linker length for a certain distance and angle between the two binding interactions against the S protein. To investigate this hypothesis, the affinity of four DRDA8 mutants were tested in which the poly‐T linker was either shortened to 5 nucleotides or extended to 20, 30, or 40 nucleotides (Figure 6B). The results indicate that the extension or shortening of the linker does not significantly improve or weaken the binding affinity. The *K*_D_ value of the original DRDA8 aptamer is 0.15 nm, while those of the T‐linker mutants range from 0.15 to 0.24 nm. Therefore, the trend observed with the poly‐T linker likely represents a simple PCR bias rather than an evolutionary change.

### 2.7 Selectivity Assessment of DRDA8 and DRDA10

**Figure 7:**
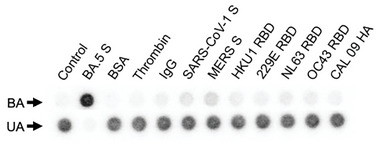
Selectivity assessment of DRDA8. Dot blot results of DRDA8 for binding to the S protein of the SARS‐CoV‐2 Omicron BA.5 variant and control proteins including BSA, thrombin, the spike proteins of SARS‐CoV‐1 and MERS, the RBD of four seasonal coronaviruses (HKU1, 229E, NL63, OC43), and the hemagglutinin (HA) protein of the A/California/04/2009 (CAL 09) influenza strain. 10 nm proteins were used in the assays. BA: bound aptamer; UA: unbound aptamer.

We finally tested the specificity of DRDA8 and DRDA10 by assessing the binding to control proteins. First, three protein targets non‐related to SARS‐CoV‐2 were tested: bovine serum albumin (BSA), human α‐thrombin, and human immunoglobulin G (IgG). BSA and IgG were chosen since they are commonly used as control proteins in biochemical assays, while thrombin was chosen as it is a popular aptamer target in the literature. **Figures** **7** and S7 (Supporting Information) show the dot blot results for DRDA8 and DRDA10, respectively. A non‐protein (i.e., buffer only) lane was used as a negative control, and the Omicron BA.5 S protein was used as a positive control. The results of the dot blot clearly indicate that neither DRD aptamer shows binding to the three non‐related control proteins.

On the same assay, DRDA8 and DRDA10 were also tested against several proteins that have varying similarity to the SARS‐CoV‐2 S protein. These included the S protein of SARS‐CoV‐1 and MERS, the receptor‐binding domain (RBD) of four different seasonal coronaviruses (HKU1, 229E, NL63, and OC43), and the hemagglutinin A protein of the CAL 09 influenza strain. Minimal binding was observed for DRDA8 against the MERS S protein, which shares ≈50% sequence similarity with the SARS‐CoV‐2 genome.^[^ ^39^ ^]^ Aside from this binding, no cross‐reactivity was observed with the other proteins, proving that the DRD library strategy can also generate aptamers with high specificity.

## 3 Conclusion

In summary, we have presented a dual random domain aptamer library strategy that directly selects for “dimer‐like” aptamers. The DRD library takes inspiration from our previous dimeric aptamers for COVID‐19, which had two 40‐nt aptamers connected with a 20‐nt poly‐T linker.^[^ ^23^ ^]^ Similarly, our library also contains a 20‐nt poly‐T linker but contains two 25‐nt random regions. In selecting for two random regions separately and providing them enough distance to form their individual binding moieties, we were able to obtain “dimer‐like” aptamers in a single selection. Sixteen rounds were completed, and the two best aptamers (DRDA8 and DRDA10) displayed a binding affinity that outperformed our previous monomeric and dimeric aptamers for the SARS‐CoV‐2 S protein. In fact, in comparison to other published aptamers that have been directly selected for SARS‐CoV‐2 (i.e., excluding those that have been engineered post‐selection), DRDA8 and DRDA10 are the current highest‐affinity aptamers for the S protein (Table S7, Supporting Information). nCoV‐S1‐Apt1 selected by Yang et al. possesses a *K*_D_ value of 0.3 nm for the S1 protein, and the DRD aptamers exceed that by twofold for binding to the trimeric S protein.^[^ ^40^, ^41^ ^]^

In testing for its dimeric qualities, we conducted two tests, which included (1) truncation assays, and (2) scramble sequence testing. The assays suggest that both binding arms are critical for high‐affinity target recognition, and they are likely working synergistically to provide a very high binding affinity for the S protein. We also ran a competition assay to determine whether DRDA8 possessed homodimeric or heterodimeric characteristics. The clear competition in each assay suggests that the arms likely recognize the same epitope on an individual S protein subunit.

While this study highlighted the “homodimeric‐like” qualities of DRDA8, which appeared to recognize identical epitopes, this outcome may be influenced by the structural characteristics of the S protein, which could present a dominant epitope commonly targeted by aptamers. Previous studies, including our own competition studies,^[^ ^8^ ^]^ have shown that many aptamers tend to bind the same epitope, potentially explaining why bifunctional aptamers targeting multiple sites were not identified in this study. Future work could investigate the development of heterodimeric configurations to enhance binding by targeting distinct epitopes. If two DRD aptamer arms can be identified that bind different epitopes, a heterodimer could be constructed to engage multiple sites on a single subunit of the S‐protein. Furthermore, a homodimer of this DRD heterodimer could be designed to bind across multiple subunits of the trimeric S protein, maximizing binding strength. Comparing the binding characteristics of DRD aptamers with those of other monomeric SARS‐CoV‐2 aptamers could also provide valuable insights and inform future screening strategies.

The DRD library strategy provides an interesting strategy for future aptamer selections, as it is capable of generating high‐affinity dimeric aptamers in a short turnaround time. This archivable platform may prove useful in situations such as future pandemics when molecular recognition elements for diagnostics and therapeutics are rapidly required.

## Conflict of Interest

The authors declare no conflict of interest.

## Supporting information



Supporting Information

## Data Availability

The data that support the findings of this study are available in the supplementary material of this article.

## References

[1] A. D. Ellington , J. W. Szostak , Nature 1990, 346, 818. 1697402 10.1038/346818a0

[2] C. Tuerk , L. Gold , Science 1990, 249, 505. 2200121 10.1126/science.2200121

[3] M. R. Dunn , R. M. Jimenez , J. C. Chaput , Nat. Rev. Chem. 2017, 1, 76.

[4] E. M. McConnell , I. Cozma , D. Morrison , Y. Li , Anal. Chem. 2020, 92, 327. 31656066 10.1021/acs.analchem.9b04868

[5] P. Kumar Kulabhusan , B. Hussain , M. Yüce , Pharmaceutics 2020, 12, 646. 32659966 10.3390/pharmaceutics12070646 PMC7407196

[6] T. D. Pollard , Mol. Biol. Cell 2010, 21, 4061. 21115850 10.1091/mbc.E10-08-0683 PMC2993736

[7] S. Qian , D. Chang , S. He , Y. Li , Anal. Chim. Acta 2022, 1196, 339511. 35151405 10.1016/j.aca.2022.339511

[8] Z. Zhang , J. Li , R. Amini , A. Mansfield , J. Gu , J. Xia , J. D. Brennan , Y. Li , Anal. Sens. 2023, 3, 202300001.

[9] A. Brown , J. Brill , R. Amini , C. Nurmi , Y. Li , Angew. Chem., Int. Ed. 2024, 63, 202318665. 10.1002/anie.202318665 38253971

[10] J. Zhou , J. J. Rossi , Chem. Biol. 2008, 15, 644. 18635000 10.1016/j.chembiol.2008.07.004

[11] J. O. McNamara , D. Kolonias , F. Pastor , R. S. Mittler , L. Chen , P. H. Giangrande , B. Sullenger , E. Gilboa , J. Clin. Invest. 2008, 118, 376. 18060045 10.1172/JCI33365 PMC2104483

[12] Q. Hughes , B. Le , G. Gilmore , R. Baker , R. Veedu , Molecules 2017, 22, 1770. 29048375 10.3390/molecules22101770 PMC6151750

[13] H. Kuai , Z. Zhao , L. Mo , H. Liu , X. Hu , T. Fu , X. Zhang , W. Tan , J. Am. Chem. Soc. 2017, 139, 9128. 28635257 10.1021/jacs.7b04547 PMC5877791

[14] M. Mammen , S.‐K. Choi , G. M. Whitesides , Angew. Chem., Int. Ed. 1998, 37, 2754. 10.1002/(SICI)1521-3773(19981102)37:20<2754::AID-ANIE2754>3.0.CO;2-3 29711117

[15] W. Bahnan , L. Happonen , H. Khakzad , V. Kumra Ahnlide , T. de Neergaard , S. Wrighton , O. André , E. Bratanis , D. Tang , T. Hellmark , L. Björck , O. Shannon , L. Malmström , J. Malmström , P. Nordenfelt , EMBO Mol. Med. 2022, 15, 16208. 10.15252/emmm.202216208 PMC9906385 36507602

[16] Y. Li , Q. Fan , B. Zhou , Y. Shen , Y. Zhang , L. Cheng , F. Qi , S. Song , Y. Guo , R. Yan , B. Ju , Z. Zhang , iScience 2022, 25, 104431. 35607524 10.1016/j.isci.2022.104431 PMC9116965

[17] G. Vauquelin , S. J. Charlton , Br. J. Pharmacol. 2013, 168, 1771. 23330947 10.1111/bph.12106 PMC3623049

[18] K. M. Ahmad , Y. Xiao , H. T. Soh , Nucleic. Acids. Res. 2012, 40, 11777. 23042245 10.1093/nar/gks899 PMC3526301

[19] M. A. Vorobyeva , A. S. Davydova , P. E. Vorobjev , D. V. Pyshnyi , A. G. Venyaminova , Int. J. Mol. Sci. 2018, 19, 470. 29401748 10.3390/ijms19020470 PMC5855692

[20] H. Hasegawa , N. Savory , K. Abe , K. Ikebukuro , Molecules 2016, 21, 421. 27043498 10.3390/molecules21040421 PMC6273865

[21] S. Manochehry , E. M. McConnell , Y. Li , Sci. Rep. 2019, 9, 17824. 31780794 10.1038/s41598-019-54005-4 PMC6883073

[22] J. Li , Z. Zhang , J. Gu , H. D. Stacey , J. C. Ang , A. Capretta , C. D. M. Filipe , K. L. Mossman , C. Balion , B. J. Salena , D. Yamamura , L. Soleymani , M. S. Miller , J. D. Brennan , Y. Li , Nucleic. Acids. Res. 2021, 49, 7267. 34232998 10.1093/nar/gkab574 PMC8287928

[23] Z. Zhang , R. Pandey , J. Li , J. Gu , D. White , H. D. Stacey , J. C. Ang , C.‐J. Steinberg , A. Capretta , C. D. M. Filipe , K. Mossman , C. Balion , M. S. Miller , B. J. Salena , D. Yamamura , L. Soleymani , J. D. Brennan , Y. Li , Angew. Chem., Int. Ed. 2021, 60, 24266. 10.1002/anie.202110819 PMC8596624 34464491

[24] J. Li , Z. Zhang , J. Gu , R. Amini , A. G. Mansfield , J. Xia , D. White , H. D. Stacey , J. C. Ang , G. Panesar , A. Capretta , C. D. M. Filipe , K. Mossman , B. J. Salena , J. B. Gubbay , C. Balion , L. Soleymani , M. S. Miller , D. Yamamura , J. D. Brennan , Y. Li , J. Am. Chem. Soc. 2022, 144, 23465. 36520671 10.1021/jacs.2c09870 PMC9762500

[25] Y. Zhou , X. Qi , Y. Liu , F. Zhang , H. Yan , ChemBioChem 2019, 20, 2494. 31083763 10.1002/cbic.201900265

[26] L. Tang , M. Huang , M. Zhang , Y. Pei , Y. Liu , Y. Wei , C. Yang , T. Xie , D. Zhang , R. Zhou , Y. Song , J. Song , Small Methods 2023, 7, 2300327. 10.1002/smtd.202300327 37086150

[27] D. E. Huizenga , J. W. Szostak , Biochemistry 1995, 34, 656. 7819261 10.1021/bi00002a033

[28] M. N. Stojanovic , P. de Prada , D. W. Landry , J. Am. Chem. Soc. 2000, 122, 11547. 29048887 10.1021/ja0022223

[29] L. S. Green , D. Jellinek , R. Jenison , A. Ostman , C. H. Heldin , N. Janjic , Biochemistry 1996, 35, 14413. 8916928 10.1021/bi961544+

[30] L. M. Hellman , M. G. Fried , Nat. Protoc. 2007, 2, 1849. 17703195 10.1038/nprot.2007.249 PMC2757439

[31] R. Gysbers , K. Tram , J. Gu , Y. Li , Sci. Rep. 2015, 5, 11405. 26091540 10.1038/srep11405 PMC4473686

[32] J. Zhu , T. Li , J. Hu , E. Wang , Anal. Bioanal. Chem. 2010, 397, 2923. 20577724 10.1007/s00216-010-3802-9

[33] M. Liu , J. Wang , Y. Chang , Q. Zhang , D. Chang , C. Y. Hui , J. D. Brennan , Y. Li , Angew. Chem. Int. Ed. Engl. 2020, 59, 7706. 32155319 10.1002/anie.202000025

[34] M. Liu , Q. Yin , Y. Chang , Q. Zhang , J. D. Brennan , Y. Li , Angew. Chem. Int. Ed. Engl. 2019, 58, 8013. 31020784 10.1002/anie.201901192

[35] P. V. Surti , M. W. Kim , L. M. T. Phan , S. K. Kailasa , A. K. Mungray , J. P. Park , T. J. Park , Trends Anal. Chem. 2022, 157, 116736.

[36] Z. Zhang , J. Li , J. Gu , R. Amini , H. Stacey , J. Ang , D. White , C. Filipe , K. Mossman , M. Miller , B. Salena , D. Yamamura , P. Sen , L. Soleymani , J. Brennan , Y. Li , Chem. ‐ Eur. J. 2022, 28, 202200078. 10.1002/chem.202200078 PMC9015322 35084794

[37] K. H. D. Crawford , R. Eguia , A. S. Dingens , A. N. Loes , K. D. Malone , C. R. Wolf , H. Y. Chu , M. A. Tortorici , D. Veesler , M. Murphy , D. Pettie , N. P. King , A. B. Balazs , J. D. Bloom , Viruses 2020, 12, 513. 32384820 10.3390/v12050513 PMC7291041

[38] M. Kohlberger , G. Gadermaier , Biotechnol. Appl. Biochem. 2021, 69, 1771. 34427974 10.1002/bab.2244 PMC9788027

[39] R. Goyal , R. K. Gautam , H. Chopra , A. K. Dubey , R. K. Singla , R. A. Rayan , M. A. Kamal , EXCLI J. 2022, 21, 1245. 36483910 10.17179/excli2022-5355 PMC9727256

[40] G. Yang , Z. Li , I. Mohammed , L. Zhao , W. Wei , H. Xiao , W. Guo , Y. Zhao , F. Qu , Y. Huang , Sig. Transduct. Target. Ther. 2021, 6, 227. 10.1038/s41392-021-00649-6 PMC8190169 34112756

[41] R. Amini , Z. Zhang , J. Li , J. Gu , J. D. Brennan , Y. Li , Anal. Sens. 2022, 2, 202200012. 10.1002/anse.202200012 PMC9082509 35574520

