# Supplementary material for: Dimeric DNA Aptamers for the Spike Protein of SARS‐CoV‐2 Derived from a Structured Library with Dual Random Domains
Source: Small Methods. 2024 Dec 20;9(6):2401600. doi: 10.1002/smtd.202401600 (PMC12182893; doi:10.1002/smtd.202401600)
Supplement: Supplementary file 1 — Supporting Information [file SMTD-9-2401600-s001.pdf]

small methods

Supporting Information

for *Small Methods*, DOI 10.1002/smtd.202401600

Dimeric DNA Aptamers for the Spike Protein of SARS-CoV-2 Derived from a Structured Library with Dual Random Domains

*Ryan Amini, Jian Ma, Zijie Zhang, Qing Wang, Jimmy Gu, Leyla Soleymani and Yingfu Li**

Supporting Information
©Wiley-VCH 2021
69451 Weinheim, Germany

Dimeric DNA Aptamers for the Spike Protein of SARS-CoV-2 Derived from a Structured Library with Dual Random Domains

Ryan Amini,[a] Jian Ma,[a] Zijie Zhang,[a] Qing Wang,[a] Jimmy Gu,[a] Leyla Soleymani[b,c,d] and Yingfu Li*[a,c,d]

[a] Department of Biochemistry and Biomedical Sciences, McMaster University, 1280 Main Street West, Hamilton, Ontario, L8S 4K1, Canada
[b] Department of Engineering Physics, McMaster University, 1280 Main Street West, Hamilton, Ontario, L8S 4K1, Canada
[c] Michael G. DeGroote Institute of Infectious Disease Research, McMaster University 1280 Main Street West, Hamilton, Ontario, L8S 4K1, Canada
[d] School of Biomedical Engineering, McMaster University,1280 Main Street West, Hamilton, Ontario, L8S 4K1, Canada

Corresponding Author: Yingfu Li; Email: liying@mcmaster.ca.

Table of Contents

Experimental Procedures S3-6

Table S1. All synthetic oligonucleotides used in this study S7

Table S2. Concentrations of DNA and protein used during SELEX S8

Table S3. Top 50 ranking sequences in pool 16 ranked by their percentage S9

Table S4. Top 50 ranking sequences in pool 16 organized by their classes S10

Table S5. Sequences of the 8 left random domains (LRD1-LRD8) observed in the Top 50 sequences in pool 16 .... S12

Table S6. Sequences of the 11 right random domains (RRD1-RRD11) observed in the Top 50 sequences in pool 16 .... S13

Table S7. K_D values of reported aptamers for SARS-CoV-2 spike protein S14

Figure S1. Assessment of binding of enriched pools for the Omicron BA.5 S protein S15

Figure S2. Assessment of binding affinity of top DRD aptamers for the Omicron BA.5 S protein S16

Figure S3. The predicted secondary structure of DRDA8 and the binding affinity of its truncated mutants S17

Figure S4. The predicted secondary structure of DRDA10 and the binding affinity of its truncated mutants S18

Figure S5. Binding affinity of DRD aptamer mutants with loop scrambled sequences S19

Figure S6. Competition between DRDA8 Truncation 2 and DRDA8 Truncation 1 for binding to the S protein S20

Figure S7. Selectivity assessment of DRDA10 .... S21

Reference S22

Author contributions S22

Experimental Procedures

Reagents and materials

DNA oligonucleotides (DNA library, forward primer, reverse primer, reverse blocked primer) were purchased from Integrated DNA Technologies (IDT) and purified via standard 10% denaturing (8 M urea) polyacrylamide gel electrophoresis. The sequences of all the oligonucleotides used in this study are listed in Table S1. His-tagged SARS-CoV-2 spike trimer protein of the Omicron BA.5 subvariant (catalog number: SPN-C522e) were expressed from human embryonic kidney 293 cells (HEK293) and purchased from Acro Biosystems. Taq DNA polymerase was purchased from GenScript. The SARS-CoV-2 spike-pseudotyped lentivirus for the Omicron BA.5 variant (Cat. No. 78652) was purchased from BPS Biosciences Inc. HisPur Ni-NTA magnetic beads (catalog number: 88831), T4 polynucleotide kinase (PNK), and deoxyribonucleoside 5′-triphosphates (dNTPs) were purchased from Thermo Scientific (Ottawa, ON, Canada). [γ-32P]-ATP was purchased from PerkinElmer. 96-well microtiter plates (clear, polystyrene, flat bottom) were from Celltreat Inc. Nitrocellulose membranes (Cat. No. 10600125) were from GE Healthcare Inc. Nylon membranes (Cat. No. NEF994001PK) were obtained from PerkinElmer Inc (Woodbridge, ON, Canada). Sodium chloride (NaCl), potassium chloride (KCl), magnesium chloride ($MgCl_2$), HEPES (4-(2-hydroxyethyl)-1-piperazineethanesulfonic acid), sodium phosphate dibasic (Na_2HPO_4), potassium phosphate monobasic (KH_2PO_4), calcium chloride ($CaCl_2$), acetic acid (HOAc), sodium acetate (NaOAc), Tween-20, bovine serum albumin (BSA, Cat. No. A7906), human IgG (Cat. No. I4506), and thrombin were purchased from Sigma Aldrich. Milli-Q water was used for each experiment.

The S-proteins for SARS-CoV-1 and MERS, and the S-protein RBD of seasonal coronavirus NL63, 229E, MERS, OC43 and Influenza-A/California/7/09 (H1N1) hemagglutinin (HA) protein, and the control lentiviruses were obtained from Dr. Matthew Miller's lab at McMaster University. The RBD proteins were generated in Expi293 cells and contained C-terminal His-tag for purification.

Conjugation for BA.5 S protein to magnetic beads

HisPur Ni-NTA magnetic beads (5% w/v, 12.5 mg/mL) were utilized to immobilize the histidine-tagged SARS-CoV-2 Omicron BA.5 spike trimer protein (BA.5 S) of interest. The conjugation process was based on a previous report.[1] 25 μL of beads was first aliquoted and washed with 500 μL of PBST buffer (500 μL, 137 mM NaCl, 2.7 mM KCl, 10 mM Na_2HPO_4, 1.8 mM KH_2PO_4, 0.01% v/v Tween-20). Afterward, magnetic beads pellets were then resuspended in 4×PBST buffer (25 μL). BA.5 S-protein with His-tag (25 μL, 0.6 mg/mL) and water (50 μL) were mixed with magnetic beads and incubated at 4°C for 12 h. The BA.5 SP-conjugated magnetic beads were stored at 4°C and kept away from light before use.

Selection of dual random domain aptamers for BA.5 S protein

Aptamer selection was carried out by using magnetic bead-based methods that were previously reported.[1] First, the DNA library was diluted in water and selection buffer (1× SB) and heated at 90°C for 3 minutes, followed by annealing at room temperature (RT, 23°C) for 5 minutes. Then, the storage buffer of the BA.5 S protein-conjugated magnetic beads was removed, and the protein-beads were washed once with 1× SB (0.5 mL). The DNA library solution was mixed with the protein-bead pellet, and the selection reaction was incubated at RT for 2 hours, shaking at 900 rpm. After washing three times with 1× SB, the magnetic bead pellets were resuspended with 50 μL of 1× SB and heated at 90°C for 10 minutes. The 50 μL of supernatant was collected for PCR1, by adding 1× Taq buffer (50 μL, 50 mM KCl, 10 mM Tris-HCl, 1.5 mM $MgCl_2$, 1% v/v Triton X-100, pH 9.0), forward primer (25 μL, 10 μM), reverse primer (25 μL, 10 μM), Taq DNA polymerase (5 μL, 5 U/μL), dNTP (10 μL, 10 mM), and ddH_2O (335 μL). PCR1 was carried out using the following temperature profile: preheating at 94°C for 30 s; thermo cycles of 94°C for 30 s, 50°C for 30 s, and 72°C for 30 s; annealing at 72°C for 5 min. Afterwards, the PCR1 product was utilized as the template for PCR2. The PCR2 mixture was prepared by mixing the PCR1 product (100 μL), forward primer (50 μL, 10 μM), reverse primer (50 μL, 10 μM), 10× Taq buffer (100 μL), Taq DNA polymerase (10 μL, 5 U/μL), dNTP (20 μL, 10 mM), and ddH_2O (670 μL). The amplification reaction used the same temperature profile as PCR1. Following amplification, ethanol precipitation was performed with the PCR1 product to concentrate and desalt the DNA. Briefly, the PCR2 product (1 mL) was mixed with NaOAc buffer (100 μL, 3 M, pH 5.2) and ethanol (2.5 mL, -20°C), and pelleted by centrifugation at 15,000 rpm for 20 min. The pellet was washed once with 70% v/v ethanol (2.5 mL, -20°C) after discarding the supernatant. The DNA pellet was resuspended in water and the aptamer coding strand was purified by 10% urea denaturing polyacrylamide gel electrophoresis (PAGE). The gel band was visualized using the UV-shadow method, and the sense strand was cut out, and eluted using elution buffer (700 μL, 200 mM NaCl, 10 mM Tris, 1 mM EDTA, pH 7.5). Ethanol precipitation was repeated, as described above, and the enriched library was quantified by UV-Vis absorbance at 260 nm for the next round. A total of 16 rounds were completed.

Sequencing data analysis

Sample preparation and sequencing analysis were carried out according to our previously described method.[2] DNA samples from the 16-round pools were tagged via PCR using Illumina sequencing primers, followed by size purification using agarose gel and quantification based on absorbance at 260 nm. The tagged samples were pooled and sequenced in paired-end mode using an Illumina MiSeq high-throughput DNA sequencer. Sequence data processing was conducted on Ubuntu 20.04 via WSL2. Raw paired-end reads were trimmed of sequencing and library primers using cutadapt 3.4.[3] The trimmed reads were then: 1) merged into consensus reads, 2) dereplicated, and 3) clustered at 90% identity using USEARCH v11.0.667_i86linux32.[4] Custom Python scripts were used to generate sequence frequencies and ranking lists. Multiple sequence alignments were done using MUSCLE v3.8.1551, and sequence logos were generated with WebLogo 3.7.8.[5,6] The processed sequencing and cluster linkage data were stored in a MySQL 8.0.22 database. Analysis of sequence copy numbers, frequencies, cluster linkages, and data visualizations were performed using the database and Origin 2018.

Radiolabelling of DRD aptamers and enriched DNA library pools

DRD aptamers and DNA library pools were labeled with γ-[^{32}P] ATP at the 5′-end using PNK reactions according to the manufacturer's protocol. As a summary, 2 μL of 1 μM DNA aptamers were mixed with 2 μL of γ-[^{32}P] ATP, 1 μL of 10 x PNK reaction buffer A, 10 U (U: unit) of PNK and 4 μL water. The mixture was incubated at 37 °C for 20 minutes, purified by 10% denaturing PAGE, and finally concentrated using ethanol precipitation.

Dot blot binding assays with SARS-CoV-2 Omicron BA.5 spike protein

Dot blot assays were performed using a Whatman Minifold-1 96-well apparatus and a vacuum pump. Before experiments, nitrocellulose membranes and nylon membranes were incubated in dot blot binding buffer (1× SB) for 1 hour. γ-[^{32}P] labelled DRD aptamers or DNA pools (1 nM) were dissolved in the binding buffer and heated at 90 °C for 5 minutes, and then cooled at room temperature for 20 min. Omicron BA.5 S protein was dissolved and diluted in the same buffer. 5 μL of the above aptamer solution was mixed with 15 μL of spike protein with different concentrations. The mixture was incubated at room temperature for 1 hour. The dot blot apparatus was assembled with a nitrocellulose membrane on the top, a nylon membrane in the middle, and a wetted Whatman paper in the bottom. After washing each well with 100 μL of binding buffer, the binding mixtures were loaded and drained by the vacuum pump (force: 550 mmHg for 8 seconds). The wells were then washed twice with 100 μL binding buffer. The membranes were imaged using a Typhoon 9200 imager (GE Healthcare) and analyzed using Image J software.

Each binding assay was performed two times. The bound fraction (membrane-bound fraction) was quantified and plotted against the concentration of the protein. The K_D values were derived via curve fitting using Origin 8.0 using the equation: $Y = B_{max}Y/K_D +X)$ (Y is the bound fraction of the aptamer with protein, B_{max} is the maximum bound fraction of aptamer, and X is the protein concentration).

Dot blot binding assays with SARS-CoV-2 Omicron BA.5 pseudotyped lentiviruses

Dot blot assays with Omicron BA.5 SARS-CoV-2 spike-pseudotyped lentivirus and the control lentivirus were performed similarly to the procedure as described above except: the aptamer solution was diluted 1:10, and the aptamer solutions were incubated with different concentrations of virus (0 – 900 fM of viral particles) for 10 minutes rather than 1 hour.

Electrophoretic mobility shift assays (EMSA) with SARS-CoV-2 Omicron BA.5 spike protein

The binding of DRD aptamers and DNA pools with Omicron BA.5 S protein was tested by an electrophoretic mobility shift assay (EMSA). The γ-[^{32}P] labelled DRD aptamers or DNA pools (1 nM) were dissolved in the binding buffer and heated at 90 °C for 5 minutes, and then cooled at room temperature for 20 min. Omicron BA.5 S protein was dissolved and diluted in the same buffer. 5 μL of the above aptamer solution was mixed with 15 μL of spike protein with different concentrations. The mixture was incubated at room temperature for 1 hour. The samples were analyzed using miniature 10% native PAGE via the Bio-Rad Mini-PROTEAN Tetra Cell

apparatus, running the gel for 20 min at 100 V. The gels were imaged using a Typhoon 9200 imager (GE Healthcare) and analyzed using Image J software.

Statistical Analysis

Prior to analysis, all data were evaluated for completeness and quality. Quantification of the bound fraction in dot-blot binding assays was performed using ImageJ software. Images of the membranes were loaded into ImageJ, and densitometry was conducted following a standardized protocol. Using the "Circle" tool in ImageJ, a region of interest (ROI) was drawn around each blot corresponding to the membrane-bound fraction of the aptamer-protein complex. This ROI was sequentially moved across all blots to quantify intensity, with identical ROIs applied to all lanes to maintain measurement consistency. Measurements yielded integrated density values for each blot. To correct for background, a separate measurement was taken from the negative control (i.e., zero-protein sample) of the membrane, and this background intensity was subtracted from each band’s integrated density to obtain the net signal. The bound fraction (i.e., membrane-bound aptamer fraction) was quantified for each binding concentration and is reported as mean ± standard deviation (SD) based on two independent replicates for each concentration point (n = 3). Dissociation constant (K_D) values were determined by plotting the bound fraction against protein concentration and performing non-linear regression using a one-site binding equation in Origin 8.0 software. The fitting followed the equation $Y = B_{max}Y/K_D + X$ (Y is the bound fraction of the aptamer with protein, B_{max} is the maximum bound fraction of aptamer, and X is the protein concentration. Curve fitting provided R-squared values as a measure of goodness-of-fit, and 95% confidence intervals were determined for K_D values to assess the statistical reliability of binding affinity estimates. All post-analyses were conducted using GraphPad Prism 7.

Table S1. All synthetic oligonucleotides utilized in this study. Sequences are written 5′ to 3′. Abbreviations include: N_{25}: 25-nucleotide random region; T_{20}: 20-nucleotide polythymidine linker; iSP18 is the non-amplifiable, 18-atom hexa-ethylene glycol linker.

**Aptamer Selection**		
DRD (DNA Library; 109 nt)		TTACGTCAAGGTGTCACTCC-N_{25}-T_{20}-N_{25}-GAAGCATCTCTTTGGCGTG
FP (Forward Primer; 20 nt)		TTACGTCAAGGTGTCACTCC
RP1 (Reverse Primer 1; 19 nt)		CACGCCAAAGAGATGCTTC
RP2 (Reverse Blocked Primer; 39 nt)		TTTTTTTTTTTTTTTTTTTT/iSP18/CACGCCAAAGAGATGCTTC
**Aptamers Tested in Binding Experiments**		
**Name**	**Size (nt)**	
DRDA1	97	TTACGTCAAG GTGTCACTCC CACGATCCAT GTTGTTTACT GGTAGTTTTT TTTTGGAGTG AGCTGGGGGG GTAGTGTTGA AGCATCTCTT TGGCGTG
DRDA2	98	TTACGTCAAG GTGTCACTCC CACGATCCAT GTTGTTTACT GGTAGTTTTT TTTTTGGAGT GAGCTGGGGG GGTAGTGTTG AAGCATCTCT TTGGCGTG
DRDA3	97	TTACGTCAAG GTGTCACTCC TGTGGGTGGA ATGGGGAAGG GAGTGTTTTT TTTTGAATGC TTCATCTTAT TAGCTCTCGA AGCATCTCTT TGGCGTG
DRDA4	96	TTACGTCAAG GTGTCACTCC CACGATCCAT GTTGTTTACT GGTAGTTTTT TTTGGAGTGA GCTGGGGGGG TAGTGTTGAA GCATCTCTTT GGCGTG
DRDA5	96	TTACGTCAAG GTGTCACTCC TGTGGGTGGA ATGGGGAAGG GAGTGTTTTT TTTTGGGCTC CTTTTAAGTG CGTCGCGGAA GCATCTCTTT GGCGTG
DRDA6	97	TTACGTCAAG GTGTCACTCC TGTGGGTGGA ATGGGGAAGG GAGTGTTTTT TTTTTGGGCT CCTTTTAAGT GCGTCGCGGA AGCATCTCTT TGGCGTG
DRDA7	98	TTACGTCAAG GTGTCACTCC TGTGGGTGGA ATGGGGAAGG GAGTGTTTTT TTTTGTAATG CTTCATCTTA TTAGCTCTCG AAGCATCTCT TTGGCGTG
DRDA8	97	TTACGTCAAG GTGTCACTCC TGTGGGTGGA ATGGGGAAGG GAGTGTTTTT TTTTGTGAGA TGTGTGGTGT GGAATGGGGA AGCATCTCTT TGGCGTG
DRDA9	98	TTACGTCAAG GTGTCACTCC TGTGGGTGGA ATGGGGAAGG GAGTGTTTTT TTTTTGAATG CTTCATCTTA TTAGCTCTCG AAGCATCTCT TTGGCGTG
DRDA10	99	TTACGTCAAG GTGTCACTCC TGTGGGTGGA ATGGGGAAGG GAGTGTTTTT TTTTTCGGAG ATGTGTGTGT GAAACAGTGG GAAGCATCTC TTTGGCGTG
DRDA8T1	68	TTACGTCAAG GTGTCGTTTT TTTTTGTGAG ATGTGTGGTG TGGAATGGGG AAGCATCTCT TTGGCGTG
DRDA8T2	69	TTACGTCAAG GTGTCACTCC TGTGGGTGGA ATGGGGAAGG GAGTGTTTTT TTTTGTGATC TTTGGCGTG
DRDA8T3	40	TTACGTCAAG GTGTCGTTTT TTTTTGTGAT CTTTGGCGTG
DRDA8T4	84	AAGGTGTCAC TCCTGTGGGT GGAATGGGGA AGGGAGTGTT TTTTTTTGTG AGATGTGTGG TGTGGAATGG GGAAGCATCT CTTT
DRDA8T5	78	GTGTCACTCC TGTGGGTGGA ATGGGGAAGG GAGTGTTTTT TTTTGTGAGA TGTGTGGTGT GGAATGGGGA AGCATCTC
DRDA10T1	70	TTACGTCAAG GTGTCGTTTT TTTTTTCGGA GATGTGTGTG TGAAACAGTG GGAAGCATCT CTTTGGCGTG
DRDA10T2	70	TTACGTCAAG GTGTCACTCC TGTGGGTGGA ATGGGGAAGG GAGTGTTTTT TTTTTCGGAT CTTTGGCGTG
DRDA10T3	41	TTACGTCAAG GTGTCGTTTT TTTTTTCGGA TCTTTGGCGT G
DRDA10T4	86	AAGGTGTCAC TCCTGTGGGT GGAATGGGGA AGGGAGTGTT TTTTTTTTCG GAGATGTGTG TGTGAAACAG TGGGAAGCAT CTCTTT
DRDA10T5	80	GTGTCACTCC TGTGGGTGGA ATGGGGAAGG GAGTGTTTTT TTTTTCGGAG ATGTGTGTGT GAAACAGTGG GAAGCATCTC
DRDA8-5Ts	93	TTACGTCAAG GTGTCACTCC TGTGGGTGGA ATGGGGAAGG GAGTGTTTTT GTGAGATGTG TGGTGTGGAA TGGGGAAGCA TCTCTTTGGC GTG
DRDA8-20Ts	109	TTACGTCAAG GTGTCACTCC TGTGGGTGGA ATGGGGAAGG GAGTGTTTTT TTTTTTTTTT TTTTTTGTGA GATGTGTGGT GTGGAATGGG GAAGCATCTC TTTGGCGTG
DRDA8-30Ts	119	TTACGTCAAG GTGTCACTCC TGTGGGTGGA ATGGGGAAGG GAGTGTTTTT TTTTTTTTTT TTTTTTTTTT TTTTTTGTGA GATGTGTGGT GTGGAATGGG GAAGCATCTC TTTGGCGTG
DRDA8-40Ts	129	TTACGTCAAG GTGTCACTCC TGTGGGTGGA ATGGGGAAGG GAGTGTTTTT TTTTTTTTTT TTTTTTTTTT TTTTTTTTTT TTTTTTGTGA GATGTGTGGT GTGGAATGGG GAAGCATCTC TTTGGCGTG
MSA52	79	TTACGTCAAG GTGTCACTCC GTAGGGTTTG GCTCCGGGCC TGGCGTCGGT CGTCTCTCGC GAAGCATCTC TTTGGCGTG
DSA52	178	TTACGTCAAG GTGTCACTCC GTAGGGTTTG GCTCCGGGCC TGGCGTCGGT CGTCTCTCGC GAAGCATCTC TTTGGCGTGT TTTTTTTTTT TTTTTTTTTT TACGTCAAGG TGTCACTCCG TAGGGTTTGG CTCCGGGCCT GGCGTCGGTC GTCTCTCGCG AAGCATCTCT TTGGCGTG
DRDA8 L1 Scramble	97	TTACGTCAAG GTGTCACTCC GGTGAATTTG GGGAGGAGGG GAGTGTTTTT TTTTGTGAGA TGTGTGGTGT GGAATGGGGA AGCATCTCTT TGGCGTG
DRDA8 L2 Scramble	97	TTACGTCAAG GTGTCACTCC TGTGGGTGGA ATGGGGAAGG GAGTGTTTTT TTTTGTGAGA TGTGAGGGAT GAGGTGGGTT AGCATCTCTT TGGCGTG

Table S2. Concentrations of DNA and protein used during SELEX.

SELEX Round	DNA Library (nM)	BA.5 Spike Protein (nM)
1	10000	4000
2	1400	800
3	2000	1000
4	800	400
5	800	400
6	800	400
7	400	200
8	200	100
9	100	50
10	50	25
11	50	25
12	50	25
13	25	12.5
14	10	5
15	5	2.5
16	2.5	1.25

Table S3. Top 50 ranking sequences in pool 16 ranked by their percentage. Each sequence is named DRDAX, where DRDA stands for Dual Random Domain Aptamer, and X is the numeral that represents the ranking of the sequence in the round 16 pool. Each "-" represents a deletion in reference to the sequence of the original DNA library. The sequences are ranked according to their percentage in pool 16. Sequences are also color-coded to identify the ones that belong to the same family.

Rank in pool 16	Sequences (5′ – 3′)	% in pool 16
Library	NNNNNNNNNNNNNNNNNNNNNNNNNTTTTTTTTTTTTTTTTTTTTNNNNNNNNNNNNNNNNNNNNNNNNN	
DRDA1	CACGATCCATGTTGTTTACTGGTAGTTTTTTTTT------------GGAGTGAGCTGGGGGGGTAGTGTT	2.7968
DRDA2	CACGATCCATGTTGTTTACTGGTAGTTTTTTTTTT-----------GGAGTGAGCTGGGGGGGTAGTGTT	1.8595
DRDA3	TGTGGGTGGAATGGGGAAGGGAGTGTTTTTTTTT-----------G-AATGCTTCATCTTATTAGCTCTC	1.3718
DRDA4	CACGATCCATGTTGTTTACTGGTAGTTTTTTTT-------------GGAGTGAGCTGGGGGGGTAGTGTT	1.2727
DRDA5	TGTGGGTGGAATGGGGAAGGGAGTGTTTTTTTTT-------------GGGCTCCTTTTAAGTGCGTCGCG	1.2685
DRDA6	TGTGGGTGGAATGGGGAAGGGAGTGTTTTTTTTTT------------GGGCTCCTTTTAAGTGCGTCGCG	1.2213
DRDA7	TGTGGGTGGAATGGGGAAGGGAGTGTTTTTTTTT-----------GTAATGCTTCATCTTATTAGCTCTC	1.0827
DRDA8	TGTGGGTGGAATGGGGAAGGGAGTGTTTTTTTTT------------GTGAGATGTGTGGTGTGGAATGGG	1.0248
DRDA9	TGTGGGTGGAATGGGGAAGGGAGTGTTTTTTTTTT----------G-AATGCTTCATCTTATTAGCTCTC	0.9648
DRDA10	TGTGGGTGGAATGGGGAAGGGAGTGTTTTTTTTTT----------CGGAGATGTGTGTGTGAAACAGTGG	0.8987
DRDA11	TAGTCCTGAGGTGCCCGCGATGGACTTTTTTTTTT----------CGGAGATGTGTGTGTGAAACAGTGG	0.7954
DRDA12	TGTGGGTGGAATGGGGAAGGGAGTGTTTTTTTTTT-----------GTGAGATGTGTGGTGTGGAATGGG	0.7344
DRDA13	TGTGGGTGGAATGGGGAAGGGAGTGTTTTTTTTT-----------CGGAGATGTGTGTGTGAAACAGTGG	0.7240
DRDA14	TGTGGGTGGAATGGGGAAGGGAGTGTTTTTTTTTT----------GTAATGCTTCATCTTATTAGCTCTC	0.6932
DRDA15	CACGATCCATGTTGTTTACTGGTAGTTTTTTTTTTT----------GGAGTGAGCTGGGGGGGTAGTGTT	0.6830
DRDA16	TGTGGGTGGAATGGGGAAGGGAGTGTTTTTTTTT-----------GCCTTCGAATCTTACTAGCTCTCTC	0.6773
DRDA17	TAGTCCTGAGGTGCCCGCGATGGACTTTTTTTTTTT---------CGGAGATGTGTGTGTGAAACAGTGG	0.6750
DRDA18	TGTGGGTGGAATGGGGAAGGGAGTGTTTTTTTTTTT---------CGGAGATGTGTGTGTGAAACAGTGG	0.6723
DRDA19	TGTGGGTGGAATGGGGAAGGGAGTGTTTTTTTTTTT-----------GGGCTCCTTTTAAGTGCGTCGCG	0.6668
DRDA20	TGTGGGTGGAATGGGGAAGGGAGTGTTTTTTTT------------G-AATGCTTCATCTTATTAGCTCTC	0.5403
DRDA21	TAGTCCTGAGGTGCCCGCGATGGACTTTTTTTTT-----------CGGAGATGTGTGTGTGAAACAGTGG	0.5087
DRDA22	TGTGGGTGGAATGGGGAAGGGAGTGTTTTTTTTTT----------GCCTTCGAATCTTACTAGCTCTCTC	0.4930
DRDA23	ACATCCGAAGTTGTCCCGAGGTTGTTTTTTTTT--------------GTATGCTTTAAGGGGGTTGTGTC	0.4915
DRDA24	ACAGGCGGAGGTGTTCGCGACCCTGTTTTTTTTT-----------GGGGCTTCTAAGGGGGTTGTGTCTG	0.4717
DRDA25	CCAGCATCTTATTAGCTCTCGCTGGTTTTTTTTTT-----------CGGGTAAGGGGGTTGTGTCTGCCC	0.4152
DRDA26	TGTGGGTGGAATGGGGAAGGGAGTGTTTTTTTT------------GTAATGCTTCATCTTATTAGCTCTC	0.3979
DRDA27	TGTGGGTGGAATGGGGAAGGGAGTGTTTTTTTTTTT---------G-AATGCTTCATCTTATTAGCTCTC	0.3852
DRDA28	TAGTCCTGAGGTGCCCGCGATGGACTTTTTTTTTTTT--------CGGAGATGTGTGTGTGAAACAGTGG	0.3802
DRDA29	ACAGGCGGAGGTGTTCGCGACCCTGTTTTTTTTTT----------GGGGCTTCTAAGGGGGTTGTGTCTG	0.3741
DRDA30	CCAGCATCTTATTAGCTCTCGCTGGTTTTTTTTT------------CGGGTAAGGGGGTTGTGTCTGCCC	0.3675
DRDA31	TGTGGGTGGAATGGGGAAGGGAGTGTTTTTTTT--------------GGGCTCCTTTTAAGTGCGTCGCG	0.3658
DRDA32	TGTGGGTGGAATGGGGAAGGGAGTGTTTTTTTTTTTT--------CGGAGATGTGTGTGTGAAACAGTGG	0.3655
DRDA33	TGTGGGTGGAATGGGGAAGGGAGTGTTTTTTTT-------------GTGAGATGTGTGGTGTGGAATGGG	0.3603
DRDA34	TGTGGGTGGAATGGGGAAGGGAGTGTTTTTTTTTTT----------GTGAGATGTGTGGTGTGGAATGGG	0.3260
DRDA35	AGTGCGTAGCTAAGATGTCTAGCACTTTTTTTTT-----------GGCTTCCTAAGGGGGTTGTGTCTGG	0.3021
DRDA36	CCAGCATCTTATTAGCTCTCGCTGGTTTTTTTTTTT----------CGGGTAAGGGGGTTGTGTCTGCCC	0.2899
DRDA37	TGTGGGTGGAATGGGGAAGGGAGTGTTTTTTTTTTT---------GTAATGCTTCATCTTATTAGCTCTC	0.2681
DRDA38	ATGTGGGTGGAATGGGGAAGTGGAGTTTTTTTTT-----------CGGAGATGTGTGTGTGAAACAGTGG	0.2492
DRDA39	AGTGCGTAGCTAAGATGTCTAGCACTTTTTTTTTT----------GGCTTCCTAAGGGGGTTGTGTCTGG	0.2481
DRDA40	TGTGGGTGGAATGGGGAAGGGAGTGTTTTTTTTTTT---------GCCTTCGAATCTTACTAGCTCTCTC	0.2423
DRDA41	TGTGGGTGGAATGGGGAAGGGAGTGTTTTTTTTTTTT----------GGGCTCCTTTTAAGTGCGTCGCG	0.2383
DRDA42	ACATCCGAAGTTGTCCCGAGGTTGTTTTTTTT---------------GTATGCTTTAAGGGGGTTGTGTC	0.2330
DRDA43	TAGTCCTGAGGTGCCCGCGATGGACTTTTTTTTT-----------GGCTTCCTAAGGGGGTTGTGTCTGG	0.2283
DRDA44	ACATCCGAAGTTGTCCCGAGGTTGTTTTTTTTTT-------------GTATGCTTTAAGGGGGTTGTGTC	0.2264
DRDA45	AGTGCGTAGCTAAGATGTCTAGCACTTTTTTTTTT----------ATGCTGGGGTATATACAGTCTAGAG	0.2243
DRDA46	ACAGGCGGAGGTGTTCGCGACCCTGTTTTTTTT------------GGGGCTTCTAAGGGGGTTGTGTCTG	0.2237
DRDA47	ATGTGGGTGGAATGGGGAAGTGGAGTTTTTTTTTT----------CGGAGATGTGTGTGTGAAACAGTGG	0.2211
DRDA48	TGTGGGTGGAATGGGGAAGGGAGTGTTTTTTTT------------GCCTTCGAATCTTACTAGCTCTCTC	0.2200
DRDA49	TAGTCCTGAGGTGCCCGCGATGGACTTTTTTTTTT----------GGCTTCCTAAGGGGGTTGTGTCTGG	0.2048
DRDA50	CACGATCGATGTTGTGTACTCGTAGTTTTTTTTT------------GGAGTGAGCTGGGGGGGTAGTGTT	0.1975

Table S4. Top 50 ranking sequences in pool 16 organized by their classes (each class member has the same color code). Each "-" represents a deletion in reference to the sequence of the original DNA library.

Rank in pool 16	Sequences (5′ – 3′)	% in pool 16
Library	NNNNNNNNNNNNNNNNNNNNNNNNNTTTTTTTTTTTTTTTTTTTTNNNNNNNNNNNNNNNNNNNNNNNNN	
**Class 1**		
DRDA1	CACGATCCATGTTGTTTACTGGTAGTTTTTTTTT------------GGAGTGAGCTGGGGGGGTAGTGTT	2.7858
DRDA2	CACGATCCATGTTGTTTACTGGTAGTTTTTTTTTT-----------GGAGTGAGCTGGGGGGGTAGTGTT	1.8595
DRDA4	CACGATCCATGTTGTTTACTGGTAGTTTTTTTT-------------GGAGTGAGCTGGGGGGGTAGTGTT	1.2727
DRDA15	CACGATCCATGTTGTTTACTGGTAGTTTTTTTTTTT----------GGAGTGAGCTGGGGGGGTAGTGTT	0.6830
DRDA50	CACGATCGATGTTGTGTACTCGTAGTTTTTTTTT------------GGAGTGAGCTGGGGGGGTAGTGTT	0.1975
	**Total % in pool 16**	**6.7985**
**Class 2**		
DRDA3	TGTGGGTGGAATGGGGAAGGGAGTGTTTTTTTTT-----------G-AATGCTTCATCTTATTAGCTCTC	1.3718
DRDA7	TGTGGGTGGAATGGGGAAGGGAGTGTTTTTTTTT-----------GTAATGCTTCATCTTATTAGCTCTC	1.0827
DRDA9	TGTGGGTGGAATGGGGAAGGGAGTGTTTTTTTTTT----------G-AATGCTTCATCTTATTAGCTCTC	0.9648
DRDA14	TGTGGGTGGAATGGGGAAGGGAGTGTTTTTTTTTT----------GTAATGCTTCATCTTATTAGCTCTC	0.6932
DRDA20	TGTGGGTGGAATGGGGAAGGGAGTGTTTTTTTT------------G-AATGCTTCATCTTATTAGCTCTC	0.5403
DRDA26	TGTGGGTGGAATGGGGAAGGGAGTGTTTTTTTT------------GTAATGCTTCATCTTATTAGCTCTC	0.4152
DRDA27	TGTGGGTGGAATGGGGAAGGGAGTGTTTTTTTTTTT---------G-AATGCTTCATCTTATTAGCTCTC	0.3979
DRDA37	TGTGGGTGGAATGGGGAAGGGAGTGTTTTTTTTTTT---------GTAATGCTTCATCTTATTAGCTCTC	0.2899
	**Total % in pool 16**	**5.7558**
**Class 3**		
DRDA5	TGTGGGTGGAATGGGGAAGGGAGTGTTTTTTTTT-------------GGGCTCCTTTTAAGTGCGTCGCG	1.2685
DRDA6	TGTGGGTGGAATGGGGAAGGGAGTGTTTTTTTTTT------------GGGCTCCTTTTAAGTGCGTCGCG	1.2213
DRDA19	TGTGGGTGGAATGGGGAAGGGAGTGTTTTTTTTTTT-----------GGGCTCCTTTTAAGTGCGTCGCG	0.6668
DRDA31	TGTGGGTGGAATGGGGAAGGGAGTGTTTTTTTT--------------GGGCTCCTTTTAAGTGCGTCGCG	0.3658
DRDA41	TGTGGGTGGAATGGGGAAGGGAGTGTTTTTTTTTTTT----------GGGCTCCTTTTAAGTGCGTCGCG	0.2383
	**Total % in pool 16**	**3.7607**
**Class 4**		
DRDA8	TGTGGGTGGAATGGGGAAGGGAGTGTTTTTTTTT------------GTGAGATGTGTGGTGTGGAATGGG	1.0248
DRDA12	TGTGGGTGGAATGGGGAAGGGAGTGTTTTTTTTTT-----------GTGAGATGTGTGGTGTGGAATGGG	0.7344
DRDA33	TGTGGGTGGAATGGGGAAGGGAGTGTTTTTTTT-------------GTGAGATGTGTGGTGTGGAATGGG	0.3603
DRDA34	TGTGGGTGGAATGGGGAAGGGAGTGTTTTTTTTTTT----------GTGAGATGTGTGGTGTGGAATGGG	0.3260
	**Total % in pool 16**	**2.4455**
**Class 5**		
DRDA10	TGTGGGTGGAATGGGGAAGGGAGTGTTTTTTTTTT----------CGGAGATGTGTGTGTGAAACAGTGG	0.8987
DRDA13	TGTGGGTGGAATGGGGAAGGGAGTGTTTTTTTTT-----------CGGAGATGTGTGTGTGAAACAGTGG	0.7240
DRDA18	TGTGGGTGGAATGGGGAAGGGAGTGTTTTTTTTTTT---------CGGAGATGTGTGTGTGAAACAGTGG	0.6723
DRDA32	TGTGGGTGGAATGGGGAAGGGAGTGTTTTTTTTTTTT--------CGGAGATGTGTGTGTGAAACAGTGG	0.3658
	**Total % in pool 16**	**2.6608**
**Class 6**		
DRDA11	TAGTCCTGAGGTGCCCGCGATGGACTTTTTTTTTT----------CGGAGATGTGTGTGTGAAACAGTGG	0.7954
DRDA17	TAGTCCTGAGGTGCCCGCGATGGACTTTTTTTTTTT---------CGGAGATGTGTGTGTGAAACAGTGG	0.6750
DRDA21	TAGTCCTGAGGTGCCCGCGATGGACTTTTTTTTT-----------CGGAGATGTGTGTGTGAAACAGTGG	0.5403
DRDA28	TAGTCCTGAGGTGCCCGCGATGGACTTTTTTTTTTTT--------CGGAGATGTGTGTGTGAAACAGTGG	0.3852
	**Total % in pool 16**	**2.3959**
**Class 7**		
DRDA16	TGTGGGTGGAATGGGGAAGGGAGTGTTTTTTTTT-----------GCCTTCGAATCTTACTAGCTCTCTC	0.6773
DRDA22	TGTGGGTGGAATGGGGAAGGGAGTGTTTTTTTTTT----------GCCTTCGAATCTTACTAGCTCTCTC	0.4930
DRDA40	TGTGGGTGGAATGGGGAAGGGAGTGTTTTTTTTTTT---------GCCTTCGAATCTTACTAGCTCTCTC	0.2423
DRDA48	TGTGGGTGGAATGGGGAAGGGAGTGTTTTTTTT------------GCCTTCGAATCTTACTAGCTCTCTC	0.2200
	**Total % in pool 16**	**1.6326**
**Class 8**		
DRDA23	ACATCCGAAGTTGTCCCGAGGTTGTTTTTTTTT--------------GTATGCTTTAAGGGGGTTGTGTC	0.4915
DRDA42	ACATCCGAAGTTGTCCCGAGGTTGTTTTTTTT---------------GTATGCTTTAAGGGGGTTGTGTC	0.2330
DRDA44	ACATCCGAAGTTGTCCCGAGGTTGTTTTTTTTTT-------------GTATGCTTTAAGGGGGTTGTGTC	0.2264
	**Total % in pool 16**	**0.9509**
**Class 9**		
DRDA24	ACAGGCGGAGGTGTTCGCGACCCTGTTTTTTTTT-----------GGGGCTTCTAAGGGGGTTGTGTCTG	0.4718
DRDA29	ACAGGCGGAGGTGTTCGCGACCCTGTTTTTTTTTT----------GGGGCTTCTAAGGGGGTTGTGTCTG	0.3741
DRDA46	ACAGGCGGAGGTGTTCGCGACCCTGTTTTTTTT------------GGGGCTTCTAAGGGGGTTGTGTCTG	0.2237
	**Total % in pool 16**	**1.0696**
**Class 10**		
DRDA25	CCAGCATCTTATTAGCTCTCGCTGGTTTTTTTTTT-----------CGGGTAAGGGGGTTGTGTCTGCCC	0.4152
DRDA30	CCAGCATCTTATTAGCTCTCGCTGGTTTTTTTTT------------CGGGTAAGGGGGTTGTGTCTGCCC	0.3675
DRDA36	CCAGCATCTTATTAGCTCTCGCTGGTTTTTTTTTTT----------CGGGTAAGGGGGTTGTGTCTGCCC	0.2899
	**Total % in pool 16**	**1.0726**

**Class 11**		
DRDA35	AGTGCGTAGCTAAGATGTCTAGCACTTTTTTTTT-----------GGCTTCCTAAGGGGGTTGTGTCTGG	0.3021
DRDA39	AGTGCGTAGCTAAGATGTCTAGCACTTTTTTTTTT----------GGCTTCCTAAGGGGGTTGTGTCTGG	0.2481
**Total % in pool 16**		**0.5502**
**Class 12**		
DRDA38	ATGTGGGTGGAATGGGGAAGTGGAGTTTTTTTTT-----------CGGAGATGTGTGTGTGAAACAGTGG	0.2492
DRDA47	ATGTGGGTGGAATGGGGAAGTGGAGTTTTTTTTTT----------CGGAGATGTGTGTGTGAAACAGTGG	0.2211
	**Total % in pool 16**	**0.4703**
**Class 13**		
DRDA43	TAGTCCTGAGGTGCCCGCGATGGACTTTTTTTTT-----------GGCTTCCTAAGGGGGTTGTGTCTGG	0.2283
DRDA49	TAGTCCTGAGGTGCCCGCGATGGACTTTTTTTTTT----------GGCTTCCTAAGGGGGTTGTGTCTGG	0.2048
	**Total % in pool 16**	**0.4331**
**Class 14**		
DRDA45	AGTGCGTAGCTAAGATGTCTAGCACTTTTTTTTTT----------ATGCTGGGGTATATACAGTCTAGAG	0.2243
	**Total % in pool 16**	**0.2243**

Table S5. Sequences of the 8 left random domains (LRD1-LRD8) observed in the Top 50 sequences in pool 16. The third column identifies the appearance of each LRD in the top 14 classes in pool 16. The fourth column provides the percentage of each LRD in the top 50 sequences in pool 16, with the percentages of the top 3 LRDs highlighted by a blue background.

LRD Name	Sequence	Observed in Class:	% in pool 16
LRD1	CACGATCCATGTTGTTTACTGGTAG	1	**6.7985**
LRD2	TGTGGGTGGAATGGGGAAGGGAGTG	2, 3, 4, 5, 7	**16.2554**
LRD3	TAGTCCTGAGGTGCCCGCGATGGAC	6, 13	**2.8290**
LRD4	ACATCCGAAGTTGTCCCGAGGTTGT	8	**0.9509**
LRD5	ACAGGCGGAGGTGTTCGCGACCCTG	9	**1.0696**
LRD6	CCAGCATCTTATTAGCTCTCGCTGG	10	**1.0726**
LRD7	AGTGCGTAGCTAAGATGTCTAGCAC	11, 14	**0.7757**
LRD8	ATGTGGGTGGAATGGGGAAGTGGAG	12	**0.4703**

Table S6. Sequences of the 11 right random domains (RRD1-RRD11) observed in the Top 50 sequences in pool 16. The third column identifies the appearance of each RRD in the top 14 classes in pool 16. The fourth column provides the percentage of each RRD in the top 50 sequences in pool 16, with the percentages of the top 3 RRDs highlighted by a blue background.

RRD Name	Sequence	Observed in Class	% in pool 16
RRD1	GGAGTGAGCTGGGGGGGTAGTGTT	1	**6.7985**
RRD2	GTAATGCTTCATCTTATTAGCTCTC	2	**5.7558**
RRD3	GGGCTCCTTTTAAGTGCGTCGCG	3	**3.7607**
RRD4	GTGAGATGTGTGGTGTGGAATGGG	4	**2.4455**
RRD5	CGGAGATGTGTGTGTGAAACAGTGG	5, 6, 12	**5.5270**
RRD6	GCCTTCGAATCTTACTAGCTCTCTC	7	**1.6326**
RRD7	GTATGCTTTAAGGGGGTTGTGTC	8	**0.9509**
RRD8	GGGGCTTCTAAGGGGGTTGTGTCTG	9	**1.0696**
RRD9	CGGGTAAGGGGGTTGTGTCTGCCC	10	**1.0726**
RRD10	GGCTTCCTAAGGGGGTTGTGTCTGG	11, 13	**0.9833**
RRD11	ATGCTGGGGTATATACAGTCTAGAG	14	**0.2243**

Table S7. K_D values of reported aptamers for the SARS-CoV-2 spike protein.

No	Identifier	Aptamer	K_D (nM)	Ref
**1**	**This work**	**DRDA8**	**0.15**	This work
2	Yang-2021	nCoV-S1-Apt1	0.33	[7]
3	Minagawa-2022	RBD-Ugu1	1.2	[8]
4	Ferreira-Bravo-2021	FANA-R8–9	1.4	[9]
5	Yang-2022	SCORe	1.73	[10]
6	Chen-2022	RBD/S-A1	1.74	[11]

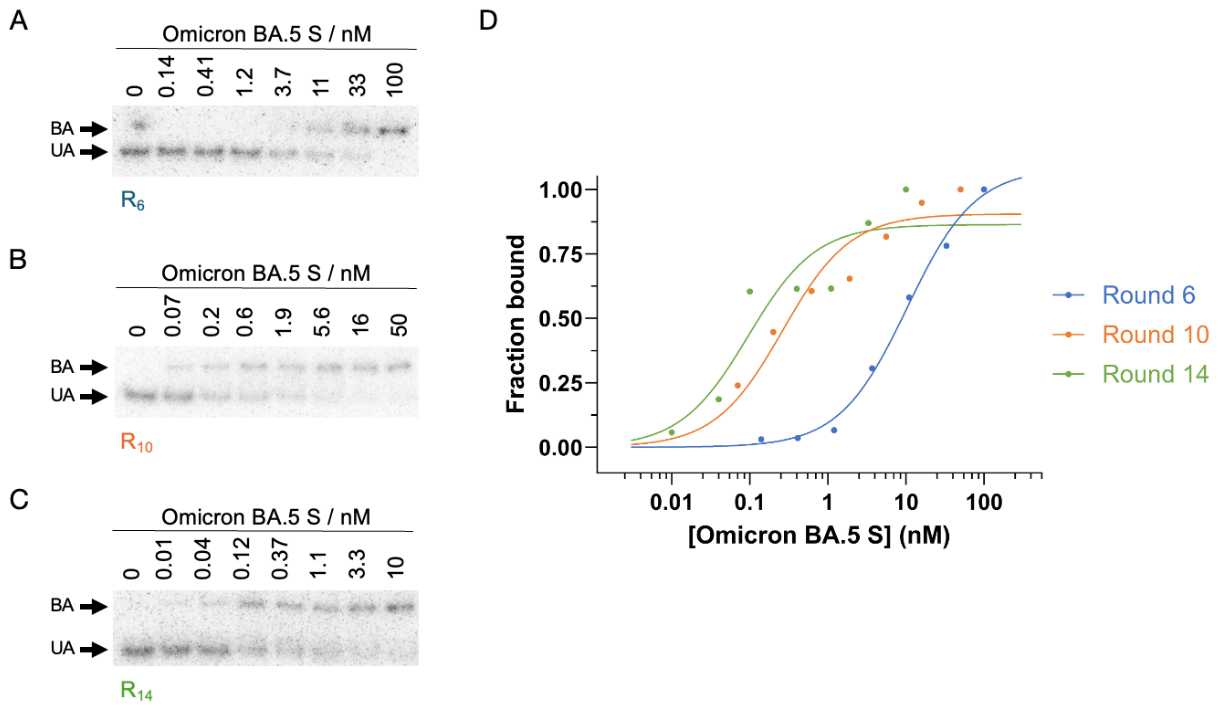

Figure S1. Assessment of binding of enriched pools for the Omicron BA.5 S protein. Representative EMSA results showing the binding of the pool of (A) round 6, (B) round 10, and (C) round 14 to the S protein. BA: bound aptamer; UA: unbound aptamer. In the assay, 100 pM radioactively labelled DNA pool was incubated with the BA.5 S protein at the indicated concentrations for 1 hour. Following incubation, the DNA-protein mixtures from each pool were subjected to native PAGE analysis to determine the binding fraction of the pool at each protein concentration, which is used to build a binding curve to estimate the K_D values of the pool. (D) Binding curves of round 6, 10, and 14 pools.

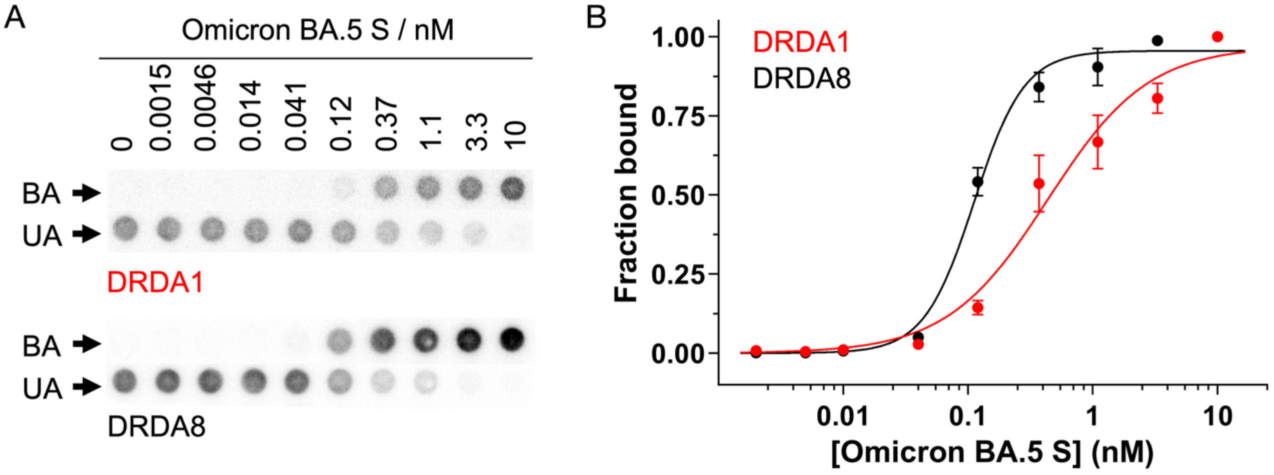

Figure S2. Assessment of binding affinity for the Omicron BA.5 S protein by DRDA1, 3, 5, 8 and 10 as the representatives for DRD aptamer classes 1, 2, 3, 4, and 5, respectively. (A) Representative dot blot results of DRDA1 and DRDA8 showing binding to the S protein. BA: bound aptamer; UA: unbound aptamer. 100 pM radioactively labelled DNA aptamer was incubated with the BA.5 S protein at concentrations ranging from 1.5-10,000 pM for 1 hour. Following incubation, the aptamer-protein mixtures were subjected dot blot analysis. (B) Binding curves of DRDA1 and DRDA8, which are used to determine their K_D values.

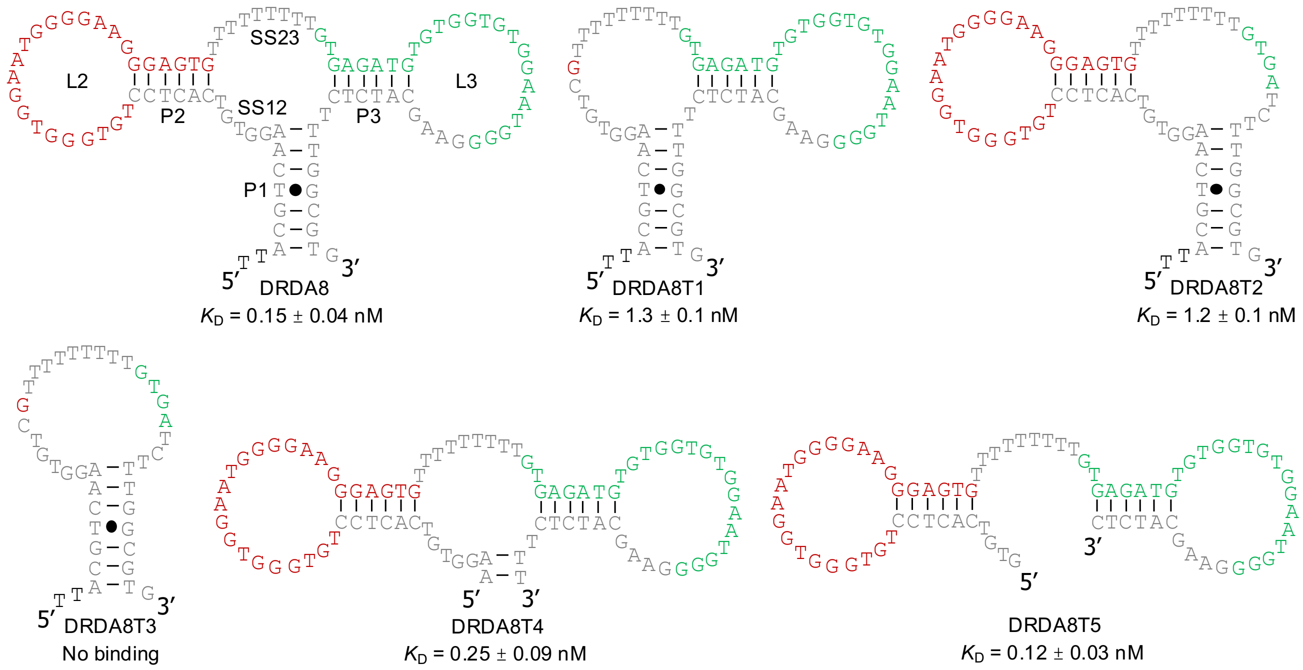

Figure S3. The predicted secondary structures and K_D values of DRDA8 and its truncated mutants. K_D values listed as mean ± SD ($n \geq 3$).

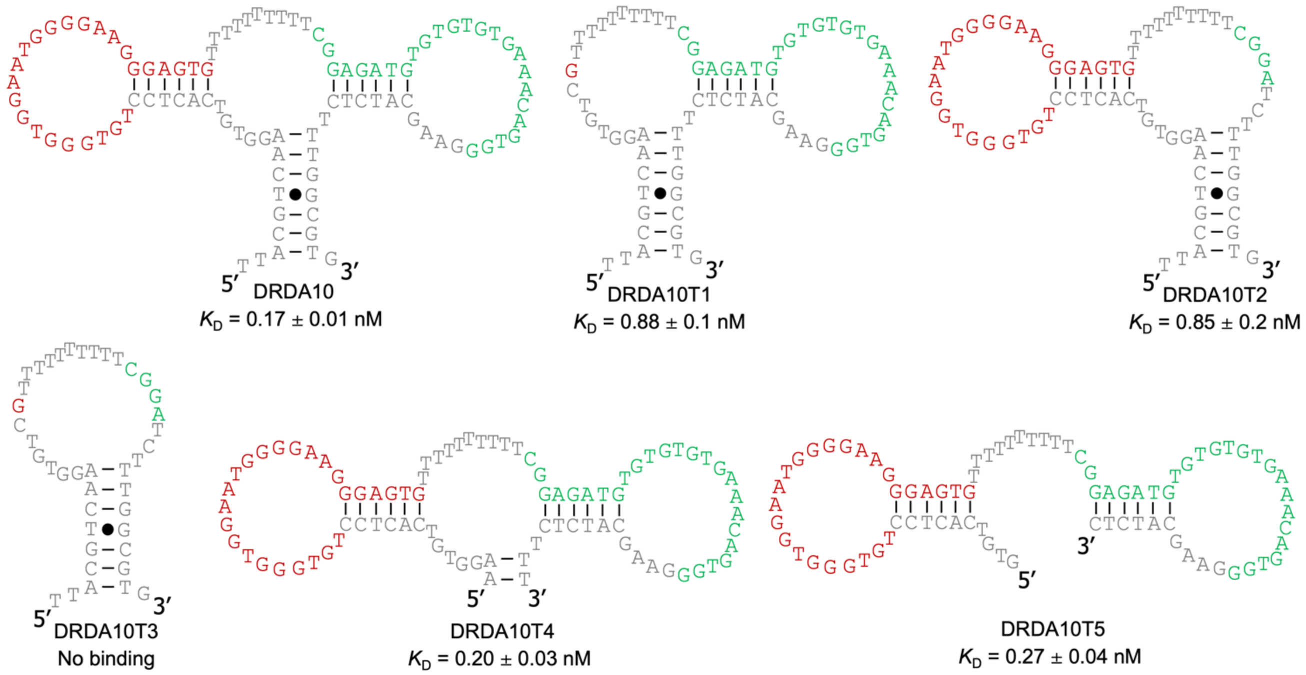

Figure S4. The predicted secondary structures and K_D values of DRDA10 and its truncated mutants. K_D values listed as mean ± SD ($n \geq 3$).

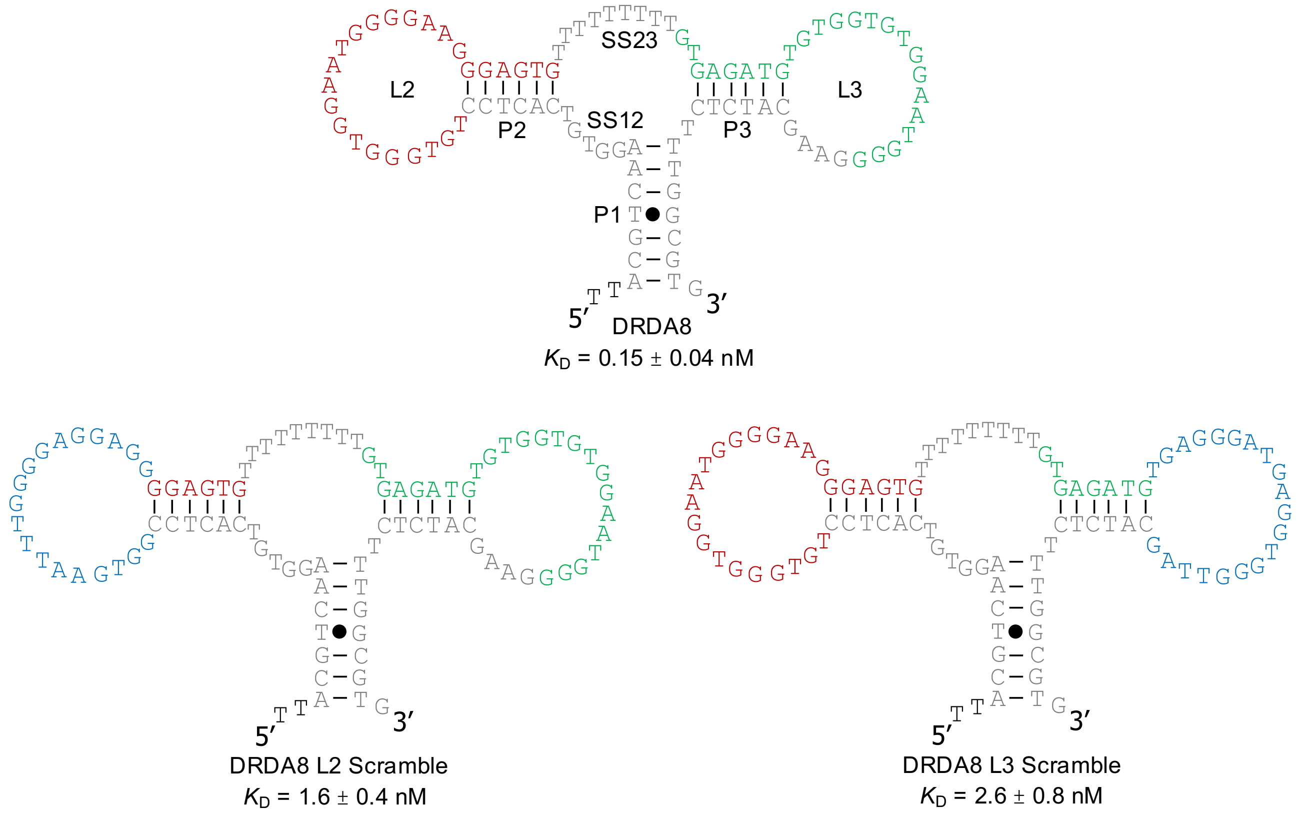

Figure S5. Binding affinity of DRDA8 mutants with loop scrambled sequences. Scrambled nucleotides are indicated in blue. K_D values listed as mean ± SD ($n \geq 3$).

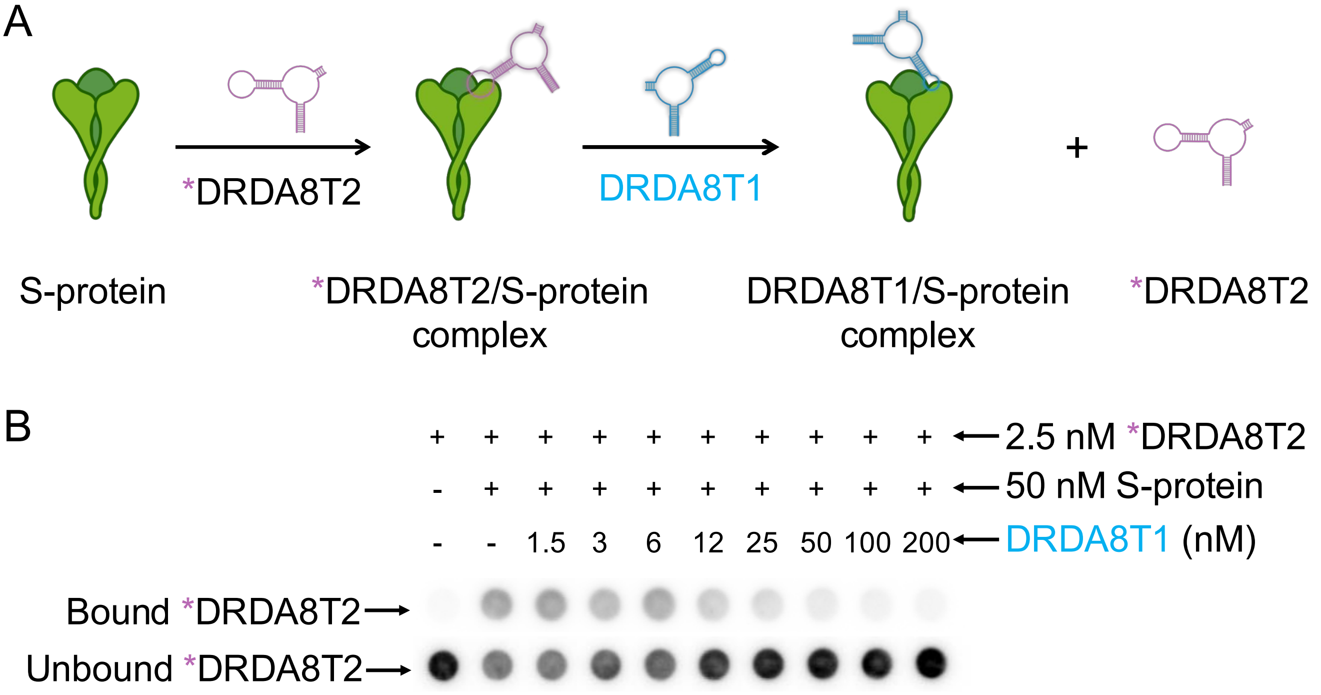

Figure S6. Competition between DRDA8T2 (DRDA8 Truncation 2) and DRDA8T1 (DRDA8 Truncation 1) for binding to the S protein. (A) Assay schematic. Radioactive Truncation 2 is allowed to bind fully to BA.5 S protein before competition with Truncation 1. (B) Assay results. A 50 nM solution of BA.5 S protein was incubated with 2.5 nM radioactive (*) DRDA8T2, followed by the addition of 1.5-200 nM non-radioactive DRDA8T1.

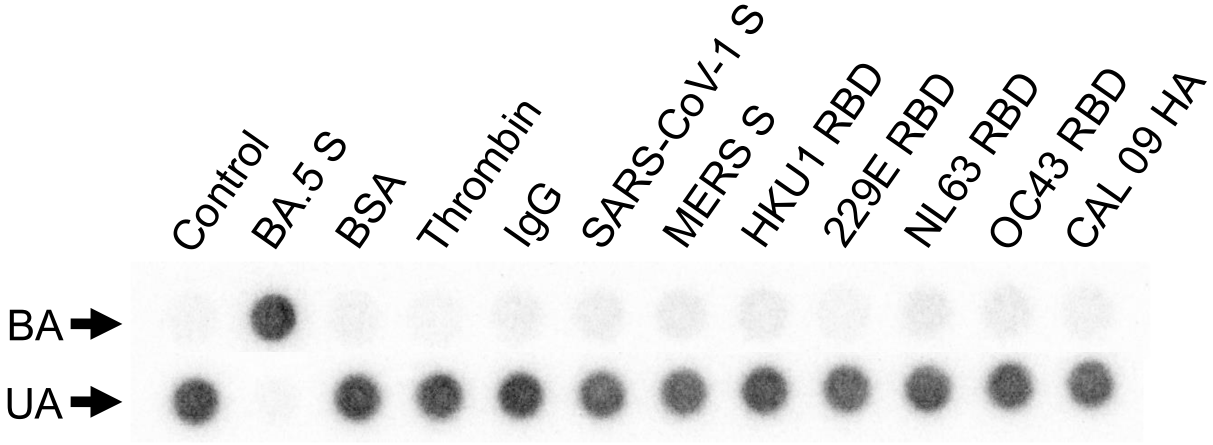

Figure S7. Selectivity assessment of DRDA10. Dot blot results of DRDA10 for binding to the S protein of the SARS-CoV-2 Omicron BA.5 variant and control proteins including BSA, thrombin, the spike proteins of SARS-CoV-1 and MERS, the RBD of four seasonal coronaviruses (HKU1, 229E, NL63, OC43), and the hemagglutinin (HA) protein of the A/California/04/2009 (CAL 09) influenza strain. 10 nM proteins were used in the assays.

References

[1] J. Li, Z. Zhang, J. Gu, H. D. Stacey, J. C. Ang, A. Capretta, C. D. M. Filipe, K. L. Mossman, C. Balion, B. J. Salena, D. Yamamura, L. Soleymani, M. S. Miller, J. D. Brennan, Y. Li, *Nucleic. Acids. Res.* **2021**, *49*, 7267–7279.
[2] R. Gysbers, K. Tram, J. Gu, Y. Li, *Sci. Rep.* **2015**, *5*, 11405.
[3] M. Martin, *EMBnet.journal* **2011**, *17*, 10-12.
[4] R. C. Edgar, *Bioinformatics* **2010**, *26*, 2460–2461.
[5] R. C. Edgar, *BMC Bioinformatics* **2004**, *5*, 113.
[6] G. E. Crooks, G. Hon, J.-M. Chandonia, S. E. Brenner, *Genome Res.* **2004**, *14*, 1188–1190.
[7] G. Yang, Z. Li, I. Mohammed, L. Zhao, W. Wei, H. Xiao, W. Guo, Y. Zhao, F. Qu, Y. Huang, *Signal Transduct. Target. Ther.* **2021**, *6*, 227–227.
[8] H. Minagawa, H. Sawa, T. Fujita, S. Kato, A. Inaguma, M. Hirose, Y. Orba, M. Sasaki, K. Tabata, N. Nomura, M. Shingai, Y. Suzuki, K. Horii, *Biochem. Biophys. Res. Commun.* **2022**, *614*, 207–212.
[9] I. Alves Ferreira-Bravo, J. J. DeStefano, *Viruses* **2021**, *13*, 1983.
[10] L. F. Yang, N. Kacherovsky, J. Liang, S. J. Salipante, S. H. Pun, *Anal. Chem.* **2022**, *94*, 12683–12690.
[11] Y. Chen, X. Yang, J. Liu, D. Zhang, J. He, L. Tang, J. Li, Q. Xiang, *Nucleosides Nucleotides Nucleic Acids* **2023**, *42*, 105–118.

Author Contributions

Y.L. and L.S. applied for the funding. R.A. and Y.L. conceived the idea. R.A. and J.M. performed the experiments and data collection. R.A., J.M., Z.Z., Q.W., J.G., and Y.L. analyzed the data. R.A. and Y.L. wrote the manuscript with contributions from all authors. Y.L. supervised and guided the project.
